# Hydrogel Adhesives for Gastrointestinal Perforation: Design Principles and Applications

**DOI:** 10.1002/EXP.20240443

**Published:** 2026-05-26

**Authors:** Yudi Pang, Shuai Tian, Qinyu Han, Yulin Deng, Jiatao Zhang, Enqiang Linghu, Qianqian Chen, Zhimin Wang

**Affiliations:** ^1^ School of Interdisciplinary Science Beijing Institute of Technology Beijing China; ^2^ School of Medical Technology Beijing Institute of Technology Beijing China; ^3^ Department of Digestive Diseases The PLA General Hospital Beijing China; ^4^ Department of Gastroenterology 970 Hospital of the PLA Joint Logistic Support Force Yantai China; ^5^ Division of Chemistry and Chemical Engineering California Institute of Technology Pasadena California USA; ^6^ MIIT Key Laboratory of Medical Molecule Science and Pharmaceutics Engineering MOE Key Laboratory of Cluster Science School of Chemistry and Chemical Engineering Beijing Institute of Technology Beijing China

**Keywords:** covalent bonding, gastrointestinal perforation, hydrogel adhesives, noncovalent interaction, wet adhesion

## Abstract

Gastrointestinal (GI) perforation, as an acute digestive condition, is difficult to heal spontaneously and requires prompt surgical intervention or bioactive adhesives to promote wound closure. Among various types of tissue adhesives, hydrogel adhesives have attracted tremendous attention and have been used in the clinic due to their atraumatic nature, good biocompatibility, and tunable physicochemical properties. Despite their promise, the bioadhesive applications with engineered hydrogels still face challenges in the wet and acidic gastric environment. This review outlines the mainstream design approaches of hydrogel adhesives through covalent and noncovalent molecular interactions, illustrating the underlying adhesive mechanisms and material properties. Representative GI applications of hydrogel adhesives are also summarized. Finally, we discuss future perspectives on the clinical translations of hydrogel adhesives in the management of GI perforations.

## Graphical abstract

There are few reviews on hydrogel adhesives that comprehensively highlight the design principles, factors affecting adhesion, and applications for gastrointestinal (GI) perforation repair. This review outlines the mainstream design approaches of hydrogel adhesives via covalent and noncovalent molecular interactions, illustrating the underlying adhesive mechanisms and properties. Representative GI applications of hydrogel adhesives are also summarized. Finally, we discuss future perspectives on clinical translations of hydrogel adhesives in the management of GI perforations.

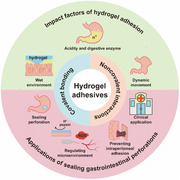

## 1 Introduction

As an acute digestive condition, gastrointestinal (GI) perforation can be caused by a variety of external or internal factors. For example, gastric and duodenal perforations are commonly caused by peptic ulcers or ingesting corrosive substances [1, 2, 3]. Small bowel perforations often result from strangulated bowel obstruction or acute appendicitis [4], while GI perforations are also caused by intestinal obstruction [5], diverticulitis [6], and inflammatory bowel disease [7]. These conditions are not only harmful to patients’ physical and mental health but also lead to mortality [8]. Extensive studies have been conducted on treating GI perforation [9, 10, 11]. Among these, sutures or staples remain the standard approach for surgical closure in clinical practice [12]. However, these complex operations are associated with a high rate of infection and tissue tearing, causing secondary injury, and leading to fluid leakage and other complications. To mitigate these issues, cyanoacrylate and human fibrin adhesives have been used as partial substitutes for sutures. However, the clinical application of cyanoacrylate has been hindered by its biotoxicity, inadequate dynamic adhesion to tissues, and the risk of postoperative adhesions [13, 14]. Meanwhile, human fibrin adhesive, a blood‐derived product, carries a potential risk of pathogen transmission, further limiting its clinical application utility.

**FIGURE 1:**
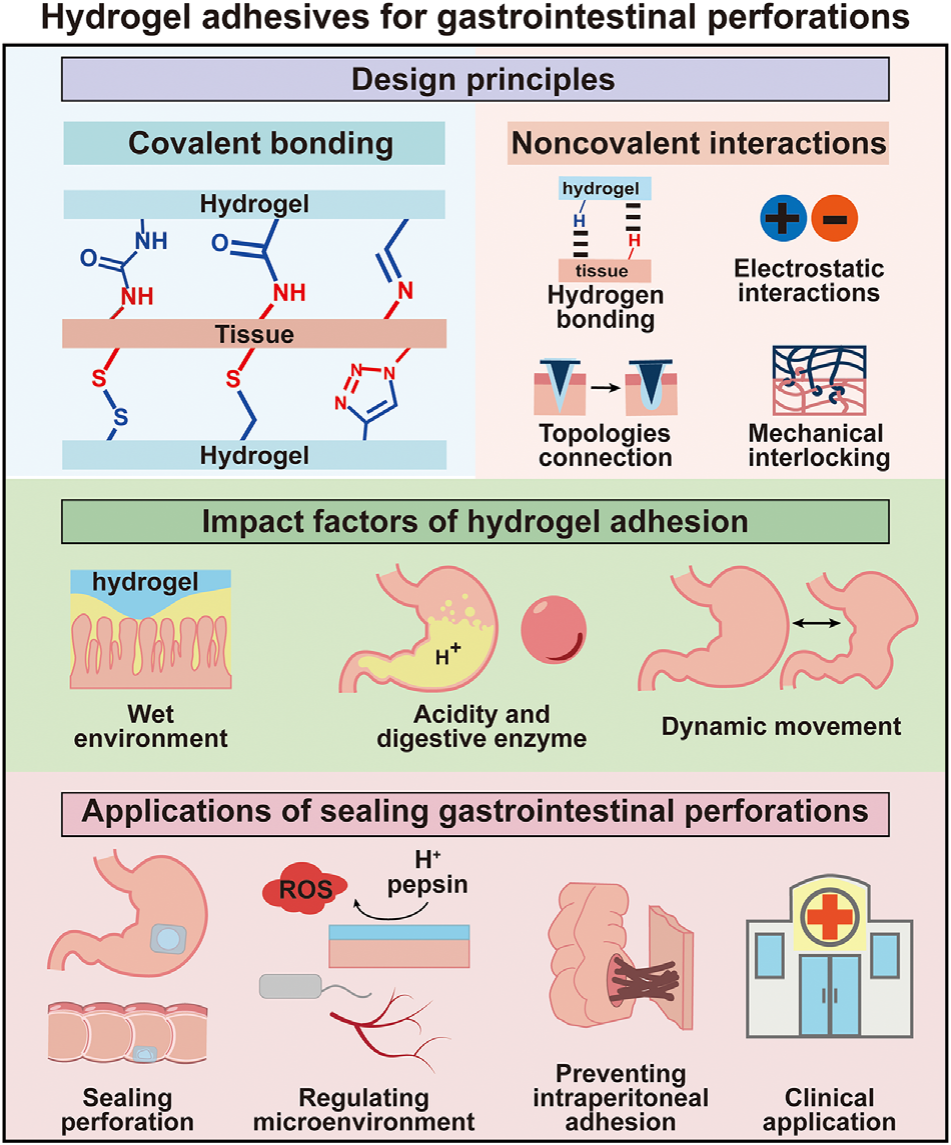
Design principles and applications of hydrogel adhesives for GI perforation repair.

The development of adhesives for GI perforation repair, such as foams, omental patches, and hydrogels, has been extensively pursued over the past decades [15, 16, 17]. Among them, hydrogels are considered promising adhesives for tissue repair due to their easily tunable physicochemical properties, high similarity to natural tissues, and good biocompatibility [18]. Hydrogels can adhere to tissues via covalent or noncovalent interactions such as amide bonds, Schiff base bonds, disulfide bonds, hydrogen bonding, and electrostatic interactions. These hydrogel adhesives not only seal perforations but also help regulate the GI wound microenvironment, preventing tissue adhesion, peritonitis, and other complications [19, 20]. Typically, ideal adhesives designed for GI perforations should meet the following criteria: (1) strong adhesion in wet environments, (2) resistance to the harsh GI environment, (3) mechanical durability, and (4) seal integrity. Currently, various types of hydrogel adhesives have been developed and applied for therapeutic purposes [21, 22, 23]. There are some review papers focused on wet adhesives, bioadhesive hydrogels, or wound closure treatment. While these reviews mainly highlight the structural design, chemical synthesis, and development of novel adhesive materials, and their diverse biomedical applications, such as tissue engineering, wound treatment as well as flexible bioelectronics [24, 25, 26]. So far, there is a lack of a timely and comprehensive review specifically summarizing the design principles, key factors influencing adhesion, biofunctionality, and clinical translation of hydrogel adhesives on GI perforation repair. In this work, we first introduce the adhesion mechanisms of hydrogel adhesives, followed by a discussion of their design strategies and the factors affecting GI tissue adhesion. We then summarize the applications of hydrogel adhesives in various GI perforation treatments and their roles in modulating the pathophysiological microenvironment. Finally, we discuss the current clinical and potential translation challenges for advancing GI therapeutic hydrogel adhesives toward clinical translation (Figure 1).

## 2 Tissue Adhesion Principles of Hydrogel Adhesives

The mechanisms underlying hydrogel adhesion can be broadly categorized into two types: covalent and noncovalent adhesion. Tissue surfaces are rich in carboxylic acid (─COOH), amine (─NH_2_), thiol (─SH), and hydroxyl (─OH) groups because cell membranes have many kinds of proteins and glycocalyx, metabolites, and extracellular matrix [27, 28, 29]. Commonly used functional groups in hydrogels include, but are not limited to, *N*‐hydroxysuccinimide (─NHS) esters, aldehyde (─CHO), ─COOH, and catechol (Cat). In this regard, covalent or noncovalent interactions can form between tissues and the hydrogel functional groups, enabling effective tissue adhesion. Generally, adhesion based on covalent bonds is more stable and irreversible, but noncovalent interaction‐based adhesion is less stable and dynamic [24, 30]. The detailed mechanisms of hydrogel adhesion are discussed below.

### 2.1 Adhesion With Covalent Bonding

Covalent adhesion is an important adhesion mechanism in hydrogel adhesives. Hydrogels react with the tissue surface groups to form stable, selective, and durable covalent bonding adhesion. As shown in Table 1, the different covalent chemical bonding types and their properties between biological tissues and adhesive hydrogels are summarized in detail.

**TABLE 1:** Representative tissue adhesion with covalent bonding for hydrogel adhesives.

Functional groups on tissue	Functional groups in hydrogel	Product	Performance	Ref.
R_1_─NH_2_	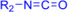	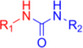	Adhesion forms instantaneously The degradation time ranges from several days to several years, and the rate is affected by the length of the alkyl chain Poor dynamic adhesion	[13, 14, 31, 32]
R_1_─NH_2_	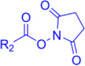	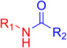	Adhesion forms within a few minutes The product is easily hydrolyzed	[33, 34, 35, 36]
R_1_─NH_2_	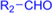	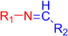	Adhesion forms within a few minutes The stability of Schiff base bonds decreases with the decrease of pH value Adhesion is dynamically reversible	[37, 38, 39, 40, 41]
R_1_─NH_2_	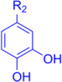	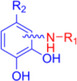	Adhesion forms within a few minutes	[23, 42, 43, 44]
R_1_─NH_2_	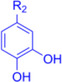	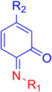		
R_1_─SH	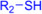	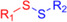	The adhesion formation time is relatively long (>5 min) Degradation is affected by reducing substances Adhesion is dynamically reversible	[45, 46]
R_1_─SH	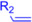	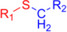	Adhesion forms within a few minutes relatively stable in acidic conditions needs additional stimulation to initiate	[47, 48, 49]
R_1_─SH	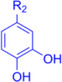	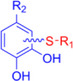	Adhesion forms within a few minutes	[23, 42, 43, 44, 50]
R_1_─N_3_	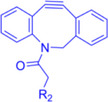	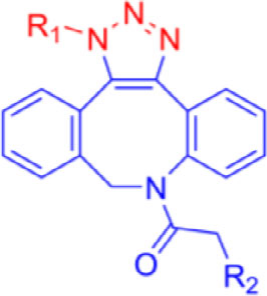	Adhesion forms instantaneously Stable in an acidic environment	[51, 52]

#### 2.1.1 ─NH_2_ and NHS Esters Reaction

**FIGURE 2:**
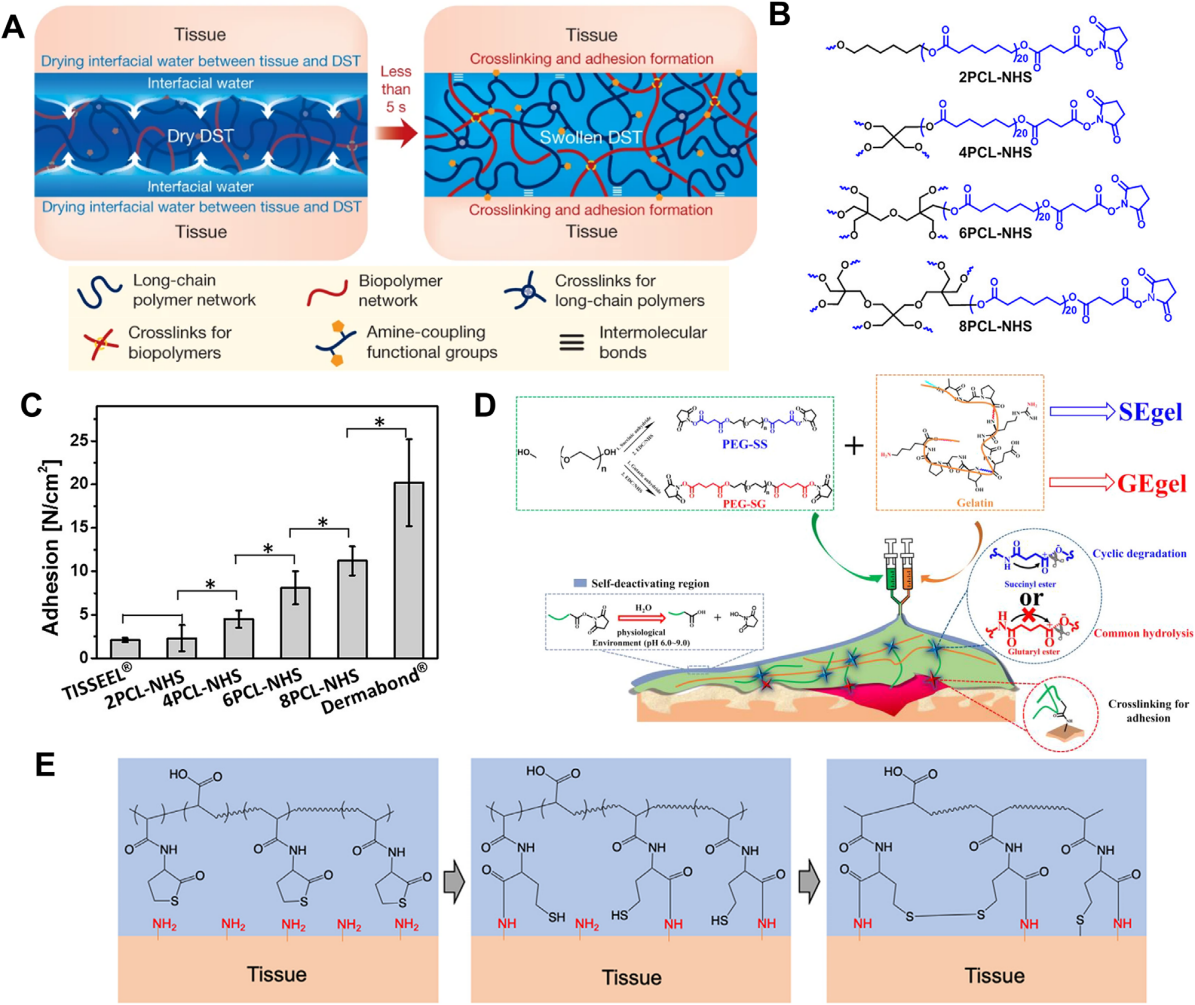
Representative hydrogel adhesives based on NHS ester. (A) Schematic illustration of the DST adhesion mechanism. DST achieved rapid adhesion by hydrating and stable adhesion through the reaction between NHS ester groups in the hydrogel and tissue–NH_2_ groups. Reproduced with permission from reference [33]. Copyright 2019, Springer Nature. (B) Chemical structures and (C) adhesion properties of NHS‐modified multiarmed PCL tissue adhesives. The more the arms of the multi‐armed PCL, the stronger the tissue adhesion strength. Reproduced with permission from reference [34]. Copyright 2020, American Chemical Society. (D) Schematic of the preparation of an injectable Janus bioadhesive, with chemical covalent bonding on one side through the reaction between NHS ester and ─NH_2_, and antiadhesion on the other side by the common hydrolysis of NHS ester. Reproduced with permission from reference [35]. Copyright 2022, American Chemical Society. (E) Design of a thiolactone‐bearing hydrogel adhesive achieving tough tissue adhesion with byproduct‐free, double crosslinking via a ring‐opening reaction. Reproduced with permission from reference [36]. Copyright 2022, Elsevier.

The biocoupling reaction between NHS esters and ─NH_2_ groups, which proceeds under mild physiological conditions, efficiently forms stable amide bonds (─CONH─), resulting in robust adhesion to tissue surfaces [53, 54, 55, 56]. Tissue adhesives based on this reaction are one of the most popular adhesive strategies that form covalent bonds with tissues [57, 58]. For example, Yuk et al. designed a dry double‐sided tape (DST) incorporating polyacrylic acid (PAA)‐g‐NHS ester and biopolymer materials. Rapid temporary adhesion was achieved due to the fast removal of interfacial water from the tissue by DST [33]. Subsequently, the PAA‐g‐NHS ester covalently crosslinked with ─NH_2_ on the tissues, and within a few minutes, the shear and tensile strength can reach more than 120 kPa towards wet porcine skins, ensuring a stable adhesion (Figure 2A). The covalent coupling reaction through NHS ester provided a more stable adhesion strategy for hydrogels to adhere to tissue surfaces. It is well established that the effective adhesion of hydrogel adhesives depends on their intrinsic strength and interfacial adhesion strength to the tissue. To further enhance the adhesion strength of NHS ester‐based tissue adhesives, Zhang et al. used NHS ester to modify the ends of arm polycaprolactone (PCL), and the chemical structures are shown in Figure 2B. Such a strategy significantly enhanced the adhesion strength, reaching up to 11.2 N cm^−2^ for the eight‐armed PCL‐NHS (Figure 2C) [34]. The adhesive could be injected by a hot glue gun, suggesting its potential as a surgical adhesive substitute.

Although NHS ester reacts with tissues in a highly reactive manner without additional stimulation [30], the biological applications of NHS ester‐based adhesives still pose certain risks. For example, NHS esters undergo hydrolysis when immersed in buffer solutions with low protein concentrations or in a physiological environment, leading to adhesion failure [57, 59, 60]. According to this feature, Wang et al. proposed a method to prepare a rapidly self‐deactivating Janus adhesive to avoid postoperative adhesion, and the schematic diagram is shown in Figure 2D [35]. Briefly, mixing poly(ethylene glycol) succinimidyl succinate with gelatin (Gel) aqueous solutions, the prepared hydrogel could be injected into the target site. This hydrogel strongly and selectively adhered to biological tissues by a covalent coupling reaction on one side, while on the other side, it lost adhesion ability due to the rapid hydrolysis of the NHS ester and the anti‐fouling ability of polyethylene glycol (PEG). The effective adhesion was validated in a cecum‐sidewall and hepatic adhesion model with rats. In addition, the biocompatibility issues are still considered due to the presence of byproducts from NHS ester aminolysis [61]. To circumvent this, Han et al. synthesized a byproduct‐free adhesive by the ring‐opening reaction between the ─NH_2_ of tissues and thiolactone groups of hydrogels to solve this problem (Figure 2E) [36]. The produced thiol termini could subsequently crosslink into disulfide bonds, enhancing the adhesion strength. This hydrogel displayed remarkable robustness and adhered firmly to both engineering substrates and wet tissues through multiple crosslinking adhesion mechanisms of thiolactone and ─SH to the tissue.

#### 2.1.2 ─NH_2_ and ─CHO Reactions

**FIGURE 3:**
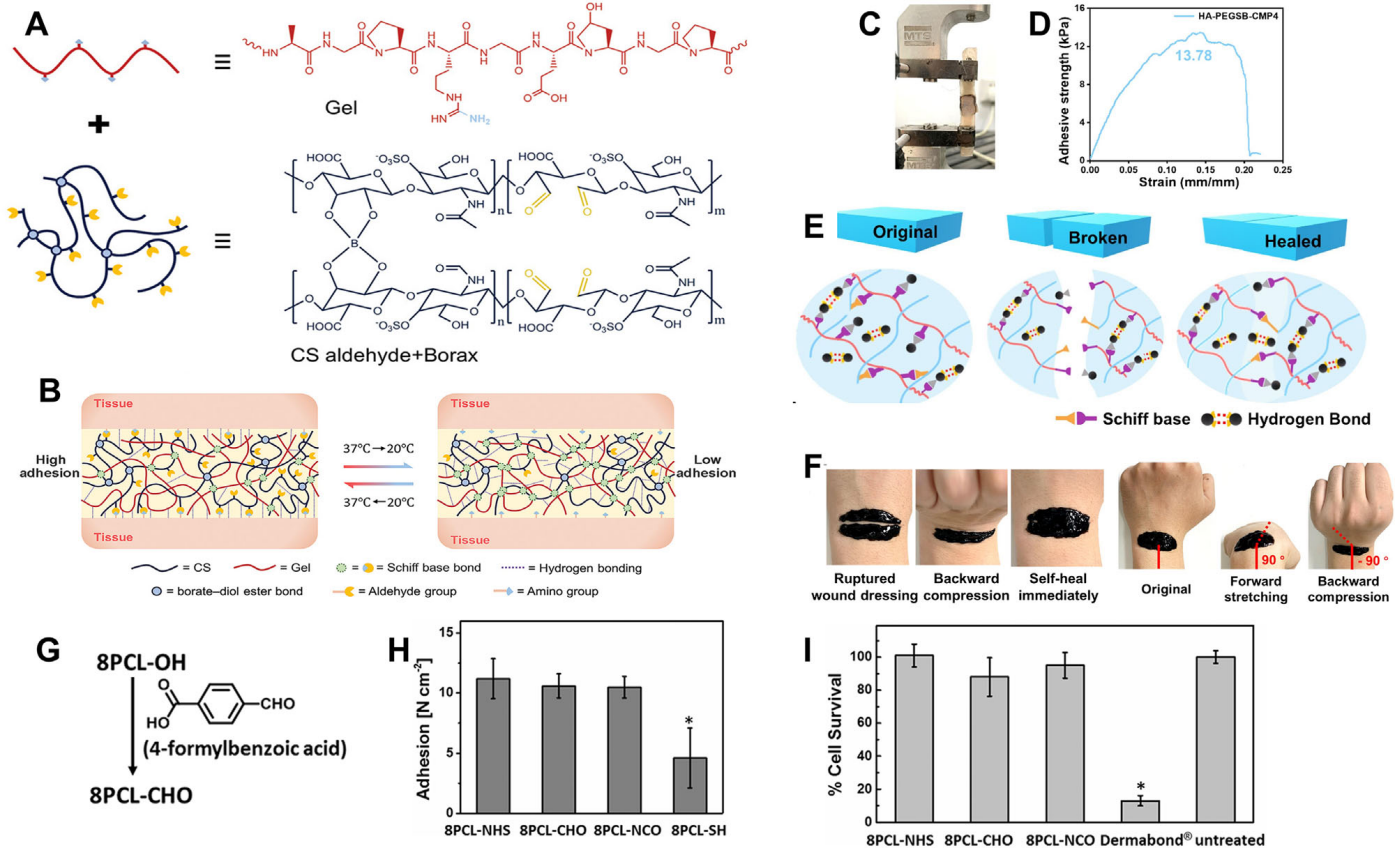
Representative hydrogel adhesives based on ─CHO groups. (A) Chemical structure of Gel and aldehyde‐modified CS with borax. (B) Mechanistic diagram of the temperature responsiveness of the injectable Gel–CS hydrogel adhesive. It achieved strong adhesion through Schiff base bonds and hydrogen bonding interactions at 37°C and reduced adhesion at 20°C due to the temperature responsiveness of Schiff base bonds and Gel. Reproduced with permission from reference [40]. Copyright 2021, John Wiley and Sons. (C) Photograph of the lap‐shear experimental setup for testing the adhesion strength of HA–PEGSB–CMP hydrogels to tissues. (D) The maximum adhesion strength of HA–PEGSB–CMP hydrogel can reach 13.78 kPa. (E) The self‐healing mechanism of HA–PEGSB–CMP hydrogel, which is crosslinked by Schiff base bonds and hydrogen bonds. (F) Photographs of self‐healing after a hydrogel is broken. HA–PEGSB–CMP hydrogel could adhere firmly to the moving wrist when applied to the skin. Reproduced with permission from reference [39]. Copyright 2021, Elsevier. (G) The synthetic route of 8‐arm PCL‐CHO. (H) The strength of the adhesive was based on the 8‐armed PCL, and the adhesion strength of 8PCL‐CHO reached 10.6 N cm^−2^. (I) Results of cytocompatibility tests by 3T3 cells. The cytocompatibility of 8PCL‐CHO was significantly higher than that of a commercial adhesive based on 2‐octyl‐cyanoacrylate, Dermabond. Reproduced with permission from reference [34]. Copyright 2020, American Chemical Society.

The Schiff base reaction, which involves the condensation of ─CHO or ketones with ─NH_2_ or ammonia, stands as a pivotal example of click chemistry. This reaction proceeds rapidly without any external stimulation or energy, producing the stable carbon─nitrogen double bond (─C═N─) linkage [62]. This spontaneous reaction allows the immediate in situ formation, finding extensive applications in tissue engineering [25, 63, 64, 65, 66]. Currently, tissue adhesives containing ─CHO groups have been widely developed [67, 68]. For example, Rana et al. prepared an injectable hydrogel adhesive within 10 s, harnessing the interaction between aldehyde sodium alginate and bioabsorbable chitosan [41]. Similarly, Kong et al. presented a similar injectable tissue adhesion combined with aldehyde‐modified oxidized guar gum and carboxymethyl chitosan for wound healing [38]. The free ─CHO group in this hydrogel enabled strong covalent adhesion to the NH_2_ of the tissue by forming ─C═N─, and the adhesive strength could reach 45 kPa. Beyond basic adhesive functionality, Schiff base‐based tissue adhesives exhibit temperature responsiveness, with broad applicability. In this regard, Zhou et al. demonstrated this with an injectable adhesive combined with Gel and chondroitin sulfate (CS) modified by aldolylation (Figure 3A) [40]. The dynamic Schiff base bonding, compounded with hydrogen bonding in the presence of borax, allowed the hydrogel to bind with tissue by both covalent and noncovalent interactions. This injectable hydrogel showed excellent tissue adhesion at 37°C, while weakening at 20°C due to the inherent thermal sensitivity of Gel and Schiff base bond breaking (Figure 3B).

Other than its temperature sensitivity, the dynamic nature of Schiff base bonds further underscores their potential in developing injectable adhesives used in dynamic wound environments. Li et al. mixed adipic dihydrazide‐modified hyaluronic acid (HA), a benzaldehyde group‐functionalized poly(ethylene glycol)‐co‐poly(glycerol sebacate) copolymer (PEGSB), and the cuttlefish melanin nanoparticle (CMP) solutions to prepare a hydrogel dressing (HA–PEGSB–CMP) to meet the need for frequent movement in the stretching area of the body [39]. This hydrogel showcased robust skin adhesion (13 kPa) through Schiff base and hydrogen bonding formation, ensuring adequate wound coverage even during wrist movement (Figure 3C,D). Additionally, its self‐healing properties, stemming from dynamic Schiff base bonds, facilitated the rapid post‐damage recovery, as represented in Figure 3E,F.

Although the ─CHO group can confer enhanced adhesion via Schiff base bond formation, excessive densities beyond the required cohesion or adhesion can cause potential biological toxicity risks, necessitating precise control over functional group ratios during the hydrogel preparation [69, 70, 71]. To mitigate this concern, Zhang et al. introduced ─CHO groups solely at the termini of the 8‐armed PCLs, which not only reduced the ─CHO density and improved the biocompatibility of hydrogel adhesives but also optimized the adhesion strength (Figure 3G–I) [34].

In the realm of gastroenterology, Schiff base bonds have the potential to be used for endoscopic GI perforation repair [72, 73]. Nevertheless, the competitive reactivity of hydrogen ions with nitrogen in Schiff bases under stomach juice poses a challenge to their stability and adhesion durability. Therefore, it is challenging to use the Schiff base reaction individually to augment their adhesion performance in this demanding environment.

#### 2.1.3 ─SH and ─SH Reaction

**FIGURE 4:**
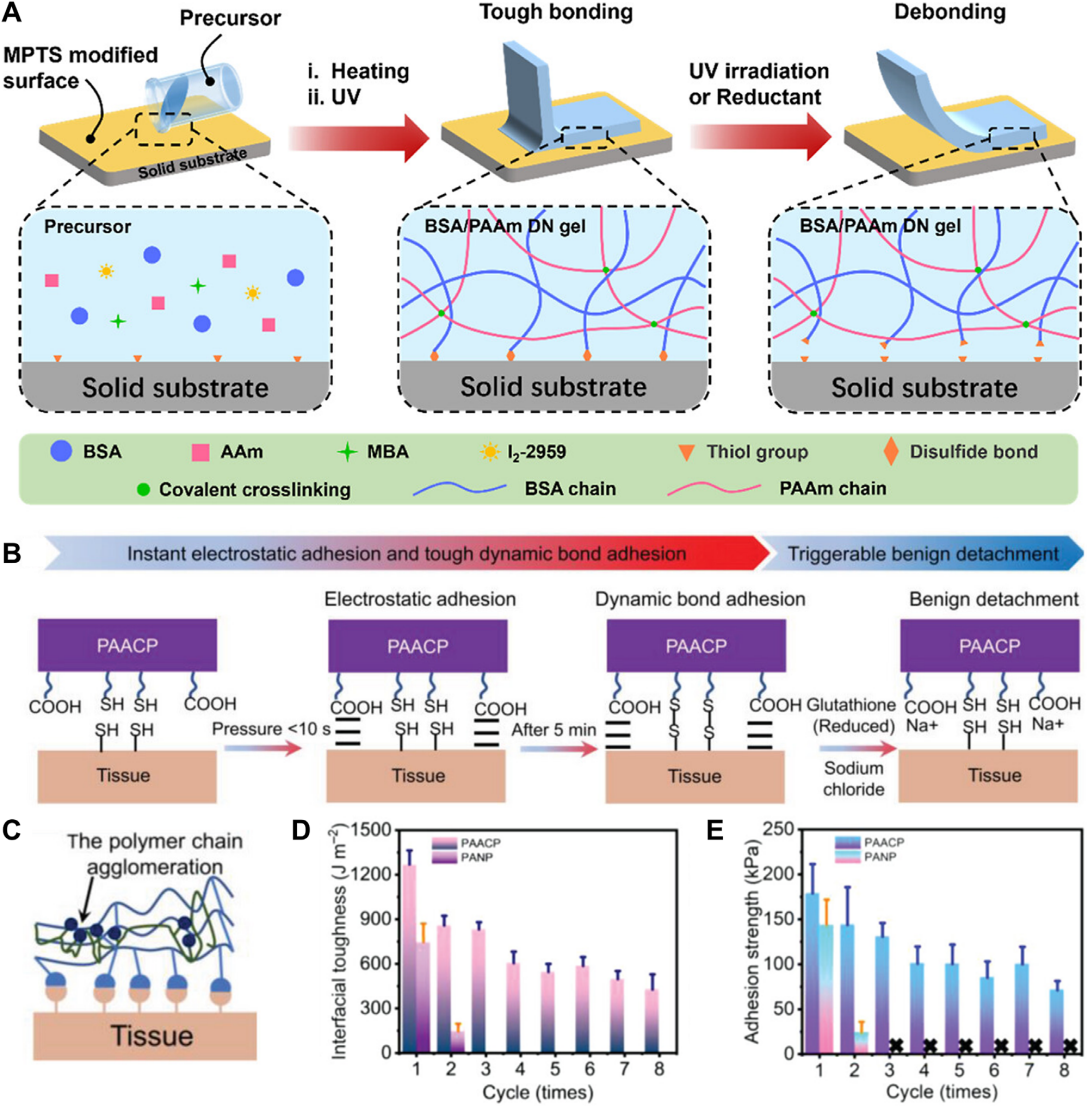
Representative hydrogel adhesives based on ─SH groups. (A) Schematic illustration of the preparation, tough bonding, and debonding of BSA/PAAm dual network gels based on ─S─S─bond. Reproduced with permission from reference [45]. Copyright 2024, American Chemical Society. (B) Schematic of the adhesion mechanism of the ─SH‐based reversible adhesion hydrogel binder PAACP. (C) The hydrophobic polymer did not compromise adhesion performance. (D) The interfacial toughness and (E) adhesion strength of PAACP subjected to 8 adhesion‐peel cycle experiments remained above 427 J m^−2^ and 70 kPa, respectively. Reproduced with permission from reference [46]. Copyright 2023, John Wiley and Sons.

The spontaneous formation of disulfide bonds (─S─S─) between ─SH groups on tissues and hydrogels without external stimuli has been documented [74, 75, 76]. Hydrogel adhesives based on these bonds demonstrate remarkable reversible adhesion properties upon disruption. Tang et al. have contributed to this field by developing a dual‐network hydrogel with tunable and robust adhesion facilitated by the ─S─S─ bond formation [45]. Figure 4A shows the straightforward one‐pot process for hydrogel synthesis, involving the dissolution of bovine serum albumin (BSA), acrylamide, Irgacure 2959, and the crosslinker MBA and initiated by heat and UV light. This hydrogel could not only adhere to processed substrates via ─S─S─ bonds but also provided efficient detachment upon the addition of glutathione or anhydrous stannous chloride. Similarly, Tian et al. fabricated a muscle sensor, PAACP, through the copolymerizing and crosslinking poly(vinyl butyral) (PVB) with acrylic acid, Gel, and chitosan‐grafted N‐acetyl‐l‐cysteine [46]. This muscle sensor achieved spontaneous adhesion rapidly, within 10 s of pressing, and its adhesive strength increased after 5 min due to the formation of dynamic ─S─S─ crosslinkers (Figure 4B). Meanwhile, the incorporation of hydrophobic PVB into the hydrogel network effectively minimized water infiltration, thereby restricting the hydrogel swelling and preserving the accessibility of functional groups at the hydrogel‐tissue interface. This strategy avoided the reduced adhesion effect due to the diffusion of adhesion groups from the highly crosslinked restricted hydrogel to the tissue interface (Figure 4C) [77]. In addition, this hydrogel boasts a high adhesion strength based on ─S─S─ covalent bonding and demonstrates resilience, enduring eight cycles of adhesion‐peeling tests (Figure 4D,E). However, the time‐consuming nature of this reaction and the potential interference from cysteine in vivo pose challenges for the practical application of ─S─S─ based robust adhesion.

#### 2.1.4 ─SH and Ene Reactions

The versatility of ─SH and ene moieties lies in their aptitude for both click chemistry and Michael addition reactions [75, 78, 79]. Both these reactions find widespread utility in tissue repair, particularly in the fabrication of hydrogels and injectable systems [80, 81, 82]. In the context of hydrogel‐based tissue adhesives, thiol‐ene chemistry emerges as a promising approach. For example, under constant UV irradiation for 120 s, thiol‐ene forms covalent bonds, endowing the hydrogel with exceptional in situ tissue adhesion capabilities [48]. In another study, Granskog et al. manufactured soft wet adhesive tissue patches combined with dendritic–linear–dendritic materials and ─SH‐containing initiators tris[2‐(3‐mercaptopropionyloxy)ethyl] isocyanurate [49]. These adhesives were initiated by 470 nm benign visible light for 30 s and chemically crosslinked by a thiol‐ene reaction, achieving a commendable adhesion strength of 21 kPa on wet pigskin. In the preparation of thiol‐ene hydrogel adhesives, although this reaction is insensitive to oxygen and has a high reaction rate, it necessitates external stimuli for initiation. Moreover, meticulous control over functional group ratios is paramount to ensure optimal hydrogel properties.

#### 2.1.5 ─SH and ─CHO Reactions

Besides the ability of ─SH on the tissue surface to link with ─SH or ene, it is also capable of reacting with ─CHO to yield thiohemiacetal linkages [83, 84]. But ─SH, typically encountered in cysteine‐rich proteins, is less prevalent in tissues compared to ─NH_2_ groups [27, 85]. Capitalizing on this chemistry, Hua et al. prepared an innovative burn wound dressing utilizing the interaction between ─SH and ─CHO to achieve skin adhesion [86]. The thiol‐aldehyde addition crosslinked hydrogel was normally prepared by mixing oxidized dextran and thiolated PEG polymer. Covalent adhesion of ─CHO to ─SH or ─NH_2_ on the skin enabled the hydrogel to adhere to the body surface as a wound dressing.

#### 2.1.6 Azide and Alkynyl Reactions

**FIGURE 5:**
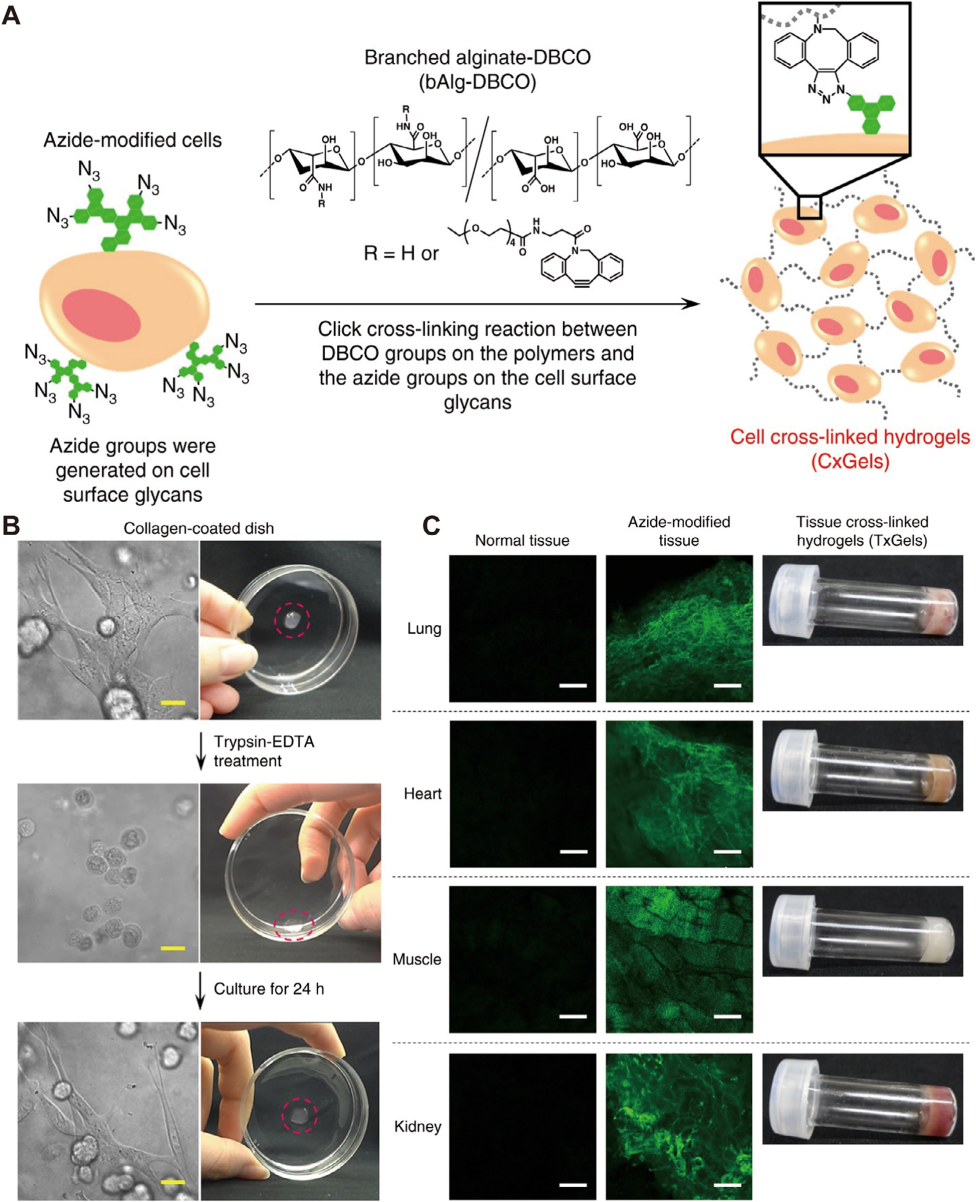
(A) Schematic illustration of CxGels crosslinked by ─N_3_ with DBCO groups. (B) Photographs of CxGels adhering to the bottom of the culture dish through cell cross‐link. It was dislodged after trypsin–EDTA treatment and adhered again after 24 h by cell attachment in culture. (C) Confocal laser scanning microscope images of azide‐modified tissue after FITC–bAlg–DBCO treatment. The N_3_‐modified tissues were able to prepare hydrogels crosslinked by the tissues with bAlg‐DBCO. Reproduced with permission from reference [52]. Copyright 2018, Springer Nature.

The azide group (─N_3_), unlike the aforementioned naturally existing functional groups, is absent in native cells and tissues but can be strategically introduced into cell membranes through metabolic glycan engineering [87, 88]. The azide groups can engage in click chemistry with alkyne groups, forming robust covalent bonds. In the most classical Copper (I)‐catalyzed Azide‐Alkyne Cycloaddition, ─N_3_ and the alkyne groups can efficiently and cleanly generate 1, 4‐disubstituted 1,2,3‐triazoles with Cu (I) as a catalyst [89]. However, the biotoxicity associated with Cu (I) poses a significant barrier to their widespread biomedical application. As an alternative, strain‐promoted alkyne‐azide cycloadditions (SPAAC) have garnered attention for their metal‐free approach [90]. In this method, the bond angle of acetylene in dibenzocyclooctyne (DBCO) undergoes a huge deformation, allowing the carbon‐carbon triple bond to open spontaneously without metal catalysts [91]. Nagahama et al. capitalized on this chemistry by integrating metabolic glycoengineering and SPAAC to prepare cell‐crosslinked hydrogels (CxGels) [52]. As shown in Figure 5A, they synthesized tetraacetylated monosaccharide *N*‐azidoacetylmannosamine (Ac_4_ManNAz) as the precursor for azide‐modified sialic acid residues and synthesized DBCO‐modified bAlg (bAlg–DBCO) from a four‐armed PEG. Following the ─N_3_ group anchoring on the cell membrane, CxGels were prepared by adding bAlg–DBCO solution (Figure 5B). Furthermore, they injected the Ac_4_ManNAz solution into the mice intraperitoneally. After the metabolic cycle, the tissues were excised carefully, shredded, and mixed with FITC‐labeled bAlg–DBCO solution, demonstrating successful metabolism to azido moieties and the formation of tissue crosslinked hydrogels (Figure 5C). This work shows the potential of SPAAC in advancing the development and preparation of tissue adhesives.

#### 2.1.7 Polyphenol‐Based Hydrogel Adhesives for Tissue Adhesion

**FIGURE 6:**
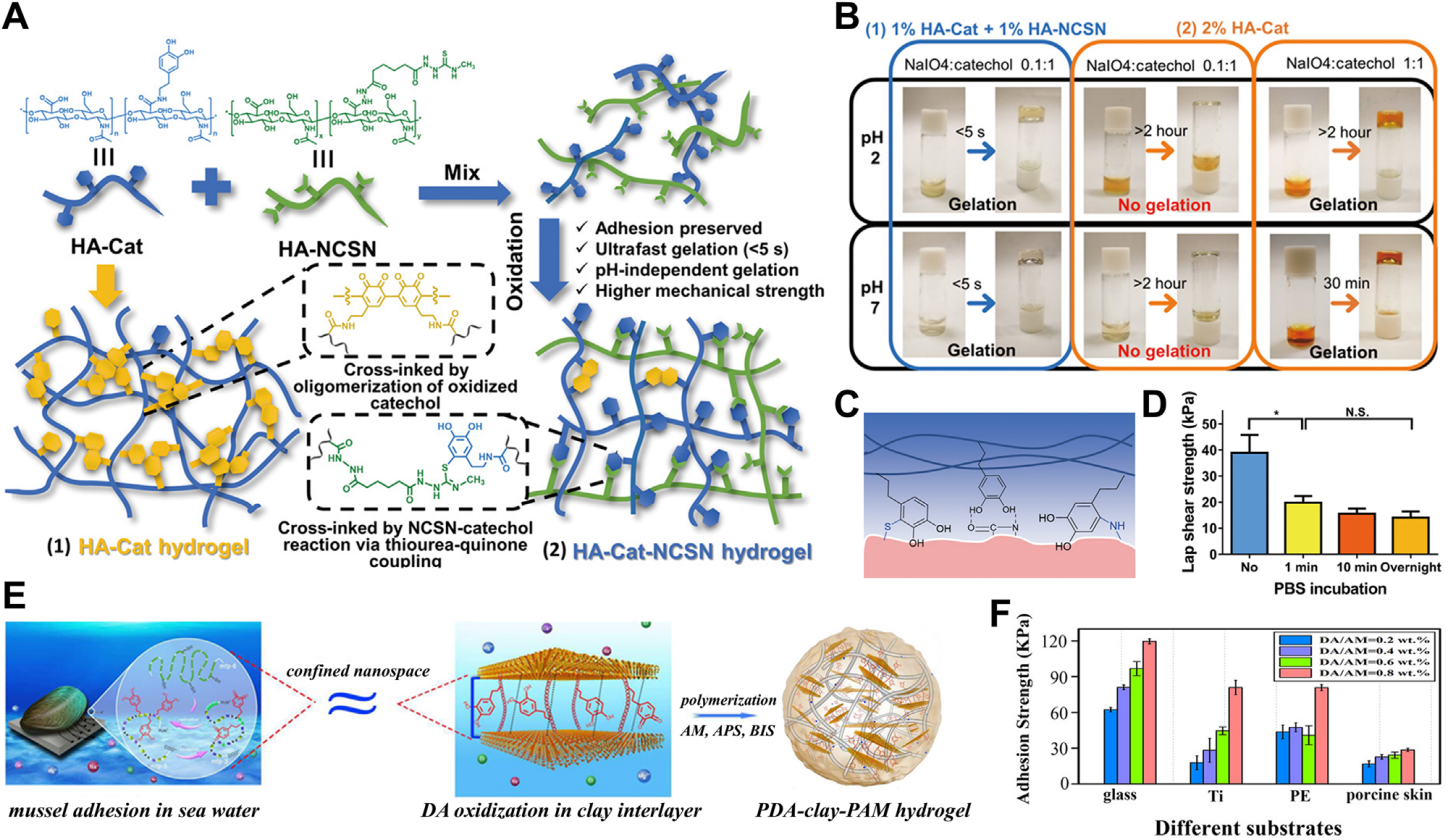
Representative Cat‐based hydrogel adhesives. (A) Design of HA‐Cat‐NCSN hydrogel. (B) After adding a small amount of oxidant, the HA‐Cat‐NCSN hydrogel formed a gel within 5 s at pH 2 and pH 7. The HA–Cat hydrogel only gelled at high doses of strong oxidant conditions, and the hydrogel showed a brown color due to Cat oxidation (NaIO_4_: Cat molar ratio). (C) Tissue adhesion mechanism of HA‐Cat‐NSCN hydrogel adhesives. (D) Adhesion strength of HA‐Cat‐NSCN hydrogels incubated in phosphate‐buffered saline solution for different times. Reproduced with permission from reference [43]. Copyright 2020, The American Association for the Advancement of Science. (E) Schematic illustration of confining space through nanoclay to prevent excessive oxidation of Cat‐based hydrogels. (F) The adhesion strength of hydrogels containing nanoconfined spaces on different substrates. Reproduced with permission from reference [109]. Copyright 2017, American Chemical Society.

In nature, mussels secrete mussel foot proteins (Mfps) rich in L‐3,4‐dihydroxyphenylalanine (DOPA), which undergo oxidation and polymerization by oxygen or oxidants, enabling robust adhesion to rocks and resilience against seawater movement through physical and chemical interactions. This mechanism inspires the design of adhesives for wet environments [42, 92, 93], including DOPA [94, 95], tannic acid [96, 97], epigallocatechin gallate [98, 99], and dihydrocaffeic acid‐based hydrogels [100, 101]. Their adhesion properties are attributed to both covalent (e.g., Schiff base bonds) and noncovalent (e.g., hydrogen bonding) interactions between their phenyl polyphenol moiety and the target tissue [23, 102, 103, 104]. To facilitate in situ oxidation of the Cat group, adding additional oxidants is often required. However, excess oxidants can compromise biocompatibility and potentially weaken the adhesion [105, 106]. Many strategies have been explored to mitigate this issue. For example, the addition of protective groups can retard the oxidation of the Cat groups under neutral or alkaline conditions. Kan et al. achieved DOPA‐mediated reversible adhesion by utilizing a bidentate between borate and the Cat groups, which mitigates excessive oxidation [107]. Furthermore, inspired by the thiol‐rich Mfp‐6 in Mfps, Xu et al. prepared a hydrogel adhesive by simple chemical modification of HA with a reducing thiourea group (abbreviated as NCSN, the four major atoms “nitrogen‐carbon‐sulfur‐nitrogen” in thiourea) and Cat, aiming to promote porcine gastric ulcer healing (Figure 6A) [43]. Compared to HA‐Cat hydrogels alone, HA‐Cat‐NCSN gelled rapidly without pH dependence and achieved rapid gelation within 5 s (Figure 6B). In addition, the hydrogel did not require the use of excessively high concentrations of oxidants; retained the unoxidized Cat groups, as shown in Figure 6C, and exhibited robust adhesion (∼40 kPa). Even after overnight immersion in phosphate‐buffered saline, its adhesion strength could be maintained at more than 10 kPa by the strong interactions between the Cat and the substrate (Figure 6D).

Although these methods can enhance the adhesion stability of the Cat groups, completely avoiding their transition to ortho‐benzoquinone remains challenging. To maintain a dynamic equilibrium between quinone and catechol groups, Gan et al. developed a dynamic catechol redox system based on Ag‐Lignin nanoparticles [108]. This system continuously produced catechol groups, thereby creating long‐lasting, reusable adhesive hydrogels. In another study, Han et al. constructed a nanoconfined environment that limited oxygen diffusion into the hydrogel matrix and inhibited Cat group oxidation through van der Waals forces between nanoclay (Figure 6E) [109]. This method preserved abundant free Cat groups in the hydrogel, and Figure 6F shows that the adhesion strength reached up to 28.5 kPa on the pigskin.

### 2.2 Adhesion With Noncovalent Interactions

Although adhesion through noncovalent interactions is generally weaker than that of a covalent bond, noncovalent adhesion is more flexible and reversible. To enhance the physical adhesion capability, multiple noncovalent interactions are often used synergistically. This section introduces representative strategies and noncovalent interaction mechanisms of tissue adhesion.

#### 2.2.1 Hydrogen Bonding Interactions

Hydrogen bonding is an attractive interaction that exhibits interaction energies ranging from 5 to 120 kJ mol^−1^, inferior to covalent bonds [110, 111]. When involving tissue adhesion, functional groups on the hydrogel surface engage in hydrogen bonding with ─NH_2_, ─COOH, ─OH, and ─SH on the tissue [112]. This interaction is particularly evident in mussel‐inspired hydrogels enriched with polyphenolic hydroxyl groups, which exhibit robust adhesion [113, 114, 115]. Hydrogel tissue adhesives based on hydrogen bonding interactions have been extensively developed [116, 117, 118].

**FIGURE 7:**
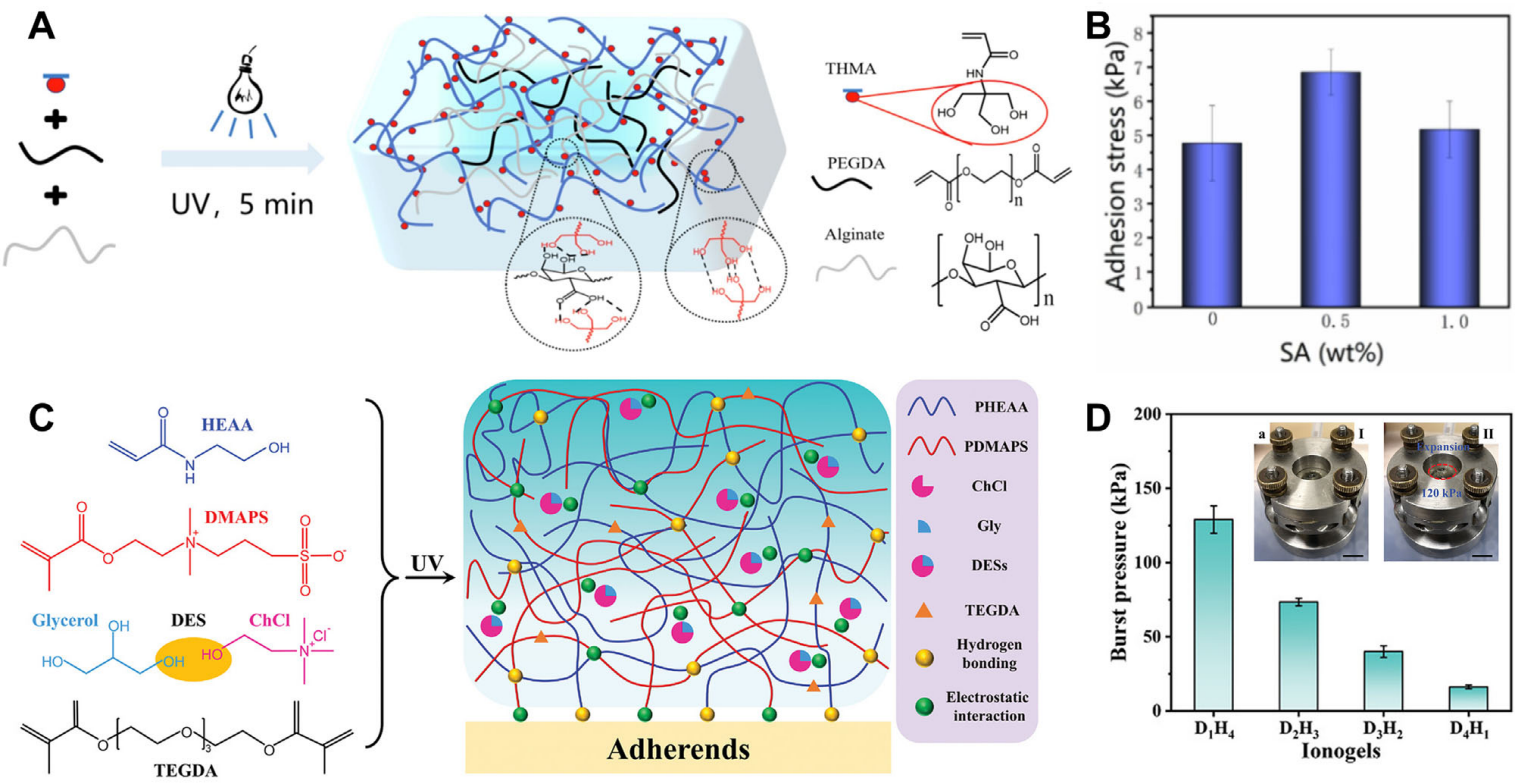
Representative hydrogel adhesives based on hydrogen bonding interactions. (A) Schematic of the preparation of THMA/PEGDA/SA hydrogels, which contained a high density of hydrogen bonds and (B) had an adhesion strength of up to 7.5 kPa. Reproduced with permission from reference [122]. Copyright 2022, Elsevier. (C) Schematic illustration and (D) burst pressure of the preparation of ionogel adhesive containing PHEAA, PDMAPS, and DES. The inserted images showed the device used to test the bursting pressure. Reproduced with permission from reference [117]. Copyright 2023 John Wiley and Sons.

Hydrogen bonding contributes to the energy dissipation, improving interfacial adhesion through dynamic and reversible conjugation‐dissociation processes [119]. However, hydrogen‐bonded hydrogels face reliability challenges for in vivo applications [120]. Increasing the number of hydrogen bonding sites and leveraging their synergistic effect can improve the adhesive strength. To maintain the long‐term adhesion and in vivo stability, Chen et al. designed a hydrogel adhesive copolymerized with *N*‐[tris(hydroxymethyl)methyl] acrylamide (THMA) and *N*‐(3‐aminopropyl)methacrylamide hydrochloride, aimed at targeting and sustaining delivery of chemotherapeutic nanodrugs [121]. This hydrogel had triple hydrogen bonding clusters on the side chain, and the adhesion originated from the high hydrogen bond density. The unique equal load sharing (ELS) configuration of THMA ensured robust adhesion to biological tissues like the kidney, spleen, and liver. Notably, even following a 30 s rinse with running water, it has still retained tissue adhesion by the ELS effect of the hydrogen‐bonding network. Based on this, Liu et al. prepared a highly stretchable and adhesive hydrogel composed of THMA, polyethylene glycol diacrylate (PEGDA), and sodium alginate (SA), achieving strong adhesion through hydrogen bonding density and energy dissipation (Figure 7A) [122]. Figure 7B shows that THMA/PEGDA/SA hydrogels' adhesion stress could reach 7.5 kPa at 0.5 wt% SA content, promising to replace sutures for effective closure of joints or moving parts wounds. Similarly, Yang et al. prepared a hydrogel combined with [(3‐Aminopropyl) methacrylamide]‐co‐THMA and oxidized methylcellulose solutions [123]. After pressing for 20 s, this hydrogel achieved stable bone adhesion under water for over 14 days due to the dense triple hydroxyl clusters and attained a wet adhesion strength of 2.32 ± 0.21 MPa.

However, hydrogen bonding adhesion is significantly influenced by environmental factors like temperature, pH, and solvents [124, 125], limiting *in viv*o applications in harsh conditions. Ge et al. overcame this by synthesizing a high‐adhesion ionogel in a deep eutectic solvent (DES) by preparing the DES from choline chloride (ChCl) and glycerol, which serve as a hydrogen bond acceptor and donor, respectively [117]. The ionogel was created through the one‐step photopolymerization of zwitterionic *N*‐(3‐sulfopropyl)‐*N*‐(methacryloxyethyl)‐*N*, *N*‐dimethylammonium betaine (DMAPS) and polarized *N*‐hydroxyethyl acrylamide (HEAA) in the DES medium (Figure 7C). This DES facilitated the formation of strong electrostatic interactions and hydrogen bonding networks within the ionogel. The resulting product exhibited ultrafast gelation and high adhesion strength, even in harsh environments such as extreme temperatures and saline solutions, sealing successfully at 120 kPa air pressure without changing its shape (Figure 7D). These advancements underscore the versatility and potential of hydrogen bonding in advancing tissue adhesion technologies.

#### 2.2.2 Electrostatic Interactions

**FIGURE 8:**
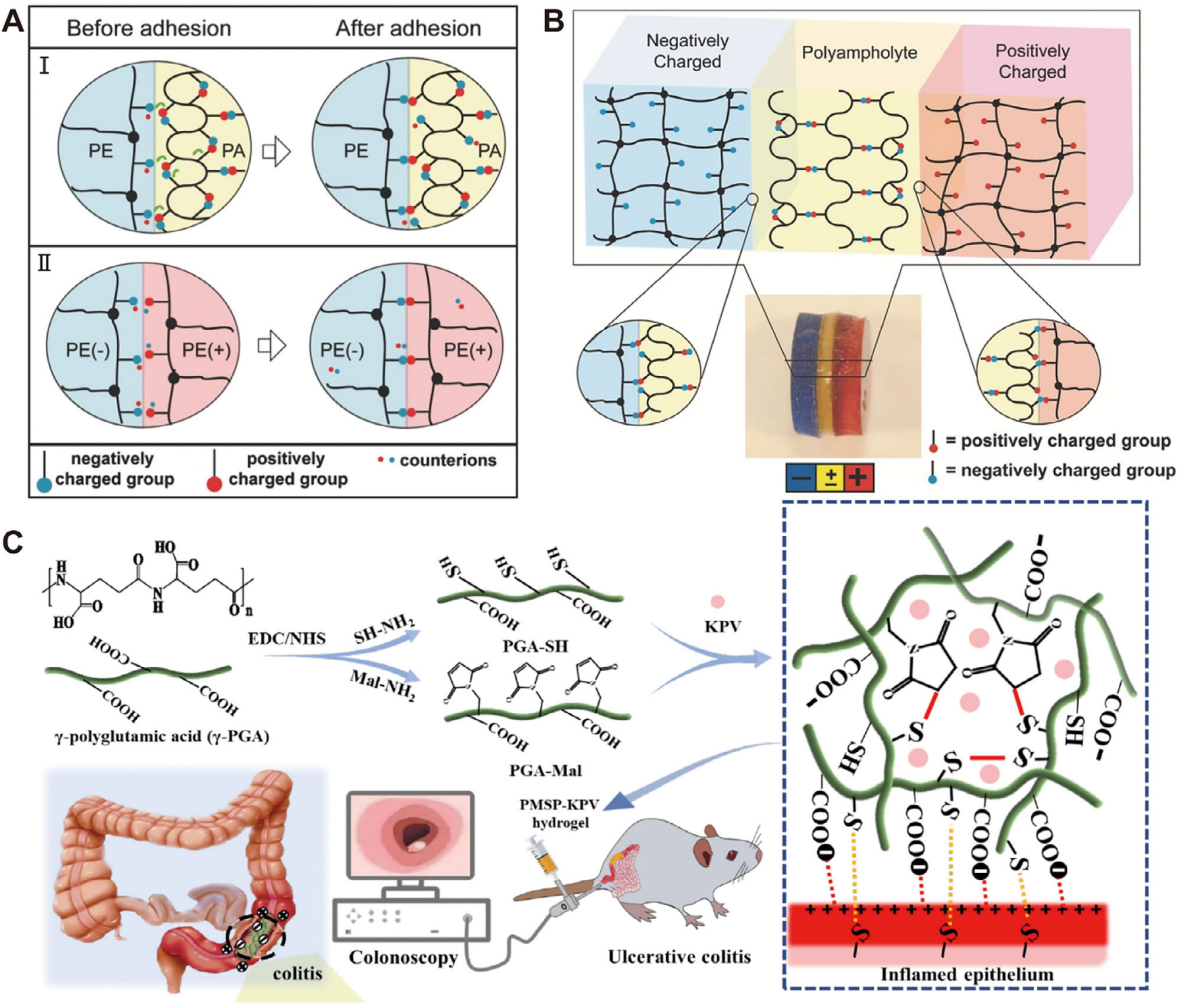
Representative hydrogel adhesives based on electrostatic interactions. (A) Electrostatic adhesion mechanism between PA and positively or negatively charged polyelectrolyte (PE) and two oppositely charged PE hydrogels. (B) Self‐regulating adhesion of PA to positively or negatively charged PE hydrogels. Reproduced with permission from reference [130]. Copyright 2015, John Wiley and Sons. (C) Schematic of the preparation of PMSP hydrogel, which adhered to the inflamed intestinal wall by electrostatic interaction. Reproduced with permission from reference [137]. Copyright 2022, Elsevier.

Electrostatic adhesion is grounded in the principle of like charges attracting and opposite charges repelling. It is generally acknowledged that tissues possess net negative charges, attributed to the phospholipid bilayers within cell membranes [126], favoring adhesion by cationic macromolecules like chitosan [127, 128], polyethyleneimine (PEI) [129], and cationic guar gum. Despite the effectiveness, these polymers possess limited adhesion strength and can lead to denaturation of tissue surfaces. To address this issue, Roy et al. developed a neutral polyampholyte (PA) hydrogel inspired by bacterial adhesion [130]. The PA hydrogel contained both polycation and polyanion segments, achieving stronger adhesion than hydrogels composed of individual charge components (Figure 8A,B). Similarly, zwitterionic hydrogels, featuring balanced positive and negative charges within the same molecule, have also been developed for tissue adhesion, relying on ion‐dipole and dipole‐dipole interactions [131, 132]. The key difference between PA hydrogels and zwitterionic hydrogels lies in their underwater adhesion capabilities, which are enhanced in the latter due to the large dipole moment of zwitterionic molecules, which can quickly hydrate through electrostatic interaction and may impede underwater adhesion [133, 134].

Furthermore, electrostatic interaction can be used to target and adhere to the inflamed colonic mucosa in ulcerative colitis (UC). Colon inflammation accompanies mucus depletion, and positively charged proteins such as transferrin, bactericidal proteins, and antimicrobial peptides accumulate [135] with the pH changes [136]. Zhao et al. utilized this unique pathological characteristic, preparing a residual carboxylate‐overloaded hydrogel constructed from maleimide γ‐polyglutamic acid, thiolated γ‐polyglutamic acid, and crosslinker thiol‐maleimide and self‐oxidized thiols, as illustrated in the schematic shown in Figure 8C [137]. This PMSP hydrogel achieved in vivo inflammatory targeting by electrostatic interactions and reached an adhesive strength of 12 ± 1.2 J·m^−2^.

While electrostatic interactions can enhance hydrogel cohesion and interfacial adhesion, their bonding energy remains significantly lower than that of chemical covalent bonds and is sensitive to ionic strength and pH variations. For example, wet or underwater environments, such as gastric fluid, weaken the electrostatic effect via the Debye screening effect, making it harder to achieve lasting adhesion [23, 138]. Aromatic groups can significantly enhance the electrostatic attraction between adjacent cationic residues and negatively charged surfaces, even under high ionic strength conditions. Gong et al. designed copolymer hydrogels, poly(cation‐adj‐π), featuring adjacent cationic‐aromatic sequences [139]. These hydrogels utilize cation–π interactions to create localized high charge density regions, effectively overcoming the Debye screening effect in high ionic‐strength environments. In this system, the aromatic moieties establish a hydrophobic microenvironment that minimizes salt‐induced electrostatic shielding, while the cationic groups create tight interfacial contact through electrostatic attraction. The poly(cation‐adj‐π) gel achieved 60 kPa adhesion strength to glass in 0.7 M NaCl solution. Furthermore, electrostatic adhesion is often complemented by hydrogen bonding and hydrophobic interactions to realize robust and enduring tissue adhesion [140, 141].

#### 2.2.3 Mechanical Interlocking and Topologies of Connection

Mechanical interlocking describes the mechanism by which, during the adhesion process between hydrogels and tissues, portions of the hydrogel function as latch‐like structures, embedding themselves into the concavities and pores present on the tissue surface [142, 143, 144, 145]. Macromolecules on the hydrogel surface can infiltrate the interstitial spaces of the tissue, establishing connective topologies through physical entanglement, even in the absence of chemically reactive functional groups [146]. However, the diffusion speed of hydrogel surface molecules into tissue constrains the overall adhesion rate. To address this limitation, Cintron‐Cruz et al. prepared a chitosan‐mediated tissue adhesion of an alginate‐polyacrylamide adhesive suture layer that accelerated the process by forming an in situ network between the hydrogel and permeable substrate, stitching them together at the molecular scale [143]. Within just a few minutes, the adhesive force exceeded 2000 J m^−2^. This chitosan‐based adhesive remained effective even in blood or Dulbecco's modified Eagle medium culture medium (pH at 7) due to chitosan's deprotonation before contact with blood‐bearing tissues, triggering an entangled network formation. The adhesion rate depended on the speed of suture network formation rather than the speed of polymer diffusion into the networks to be bonded. By adjusting the consistency of the adhesive polymer solution, the time of topology connection could be tuned from seconds to hours [146]. In another study, Ma et al. utilized ultrasound‐induced cavitation to propel and anchor primer agents into tissues, mitigating the barrier effect and achieving robust, temporally and spatially constrained adhesion between hydrogels and tissues [147]. By manipulating the distance of the ultrasound transducer to the tissue, the adhesion area and energy could be precisely controlled. The addition of pendant functional groups onto entangled polymer chains can further enhance the adhesion effect based on topological connections [144].

### 2.3 Impact Factors of Hydrogel Adhesion in GI Environment

**FIGURE 9:**
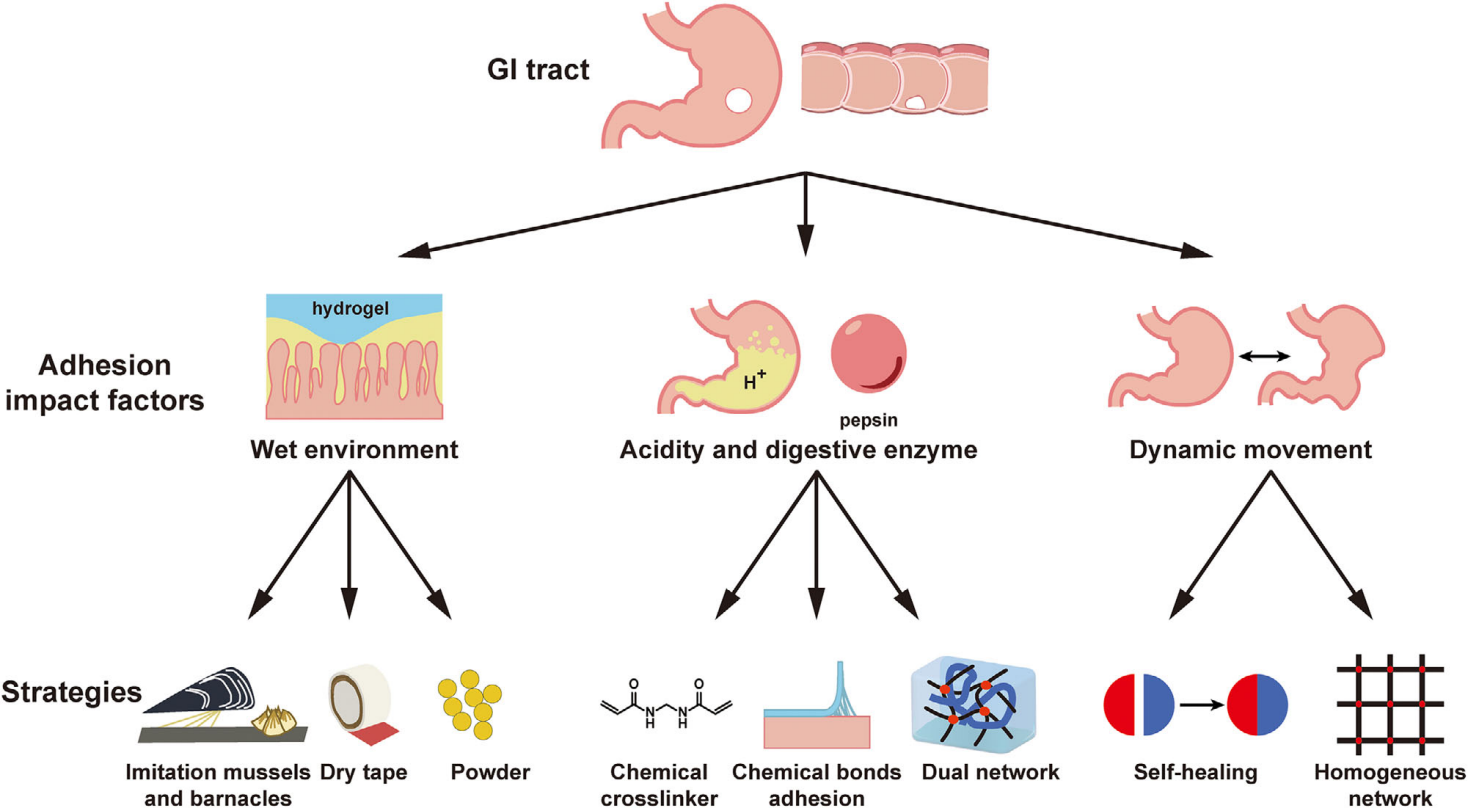
The impact factors for GI perforation repair with hydrogel adhesives and corresponding countermeasures.

GI perforation necessitates urgent and efficient closure to avoid contents leaking into the abdominal cavity. Hydrogel adhesives with good adherence can rapidly bind to the GI tract, forming a sealing barrier that supports tissue regeneration and healing. For effective GI perforation repair, these bioadhesives must adhere securely to wet GI tissues, resisting peristalsis and fluid dynamics, necessitating strong adhesion and mechanical properties [148]. This section summarizes the impact factors that hydrogel adhesives may face when adhering to GI tissues (Figure 9).

#### 2.3.1 Adhesion in a Wet Environment

A key challenge lies in adhesion within the wet GI environment, distinct from skin adhesion. The hydration layer on tissue surfaces hinders direct adhesive–substrate contact, and the water molecules compete with the substrate surface groups to interact with the adhesive functional groups [23]. For example, NHS ester‐based adhesives are prone to hydrolysis in humid environments, ultimately leading to adhesive failure. Dry gel demonstrates promising wet adhesion capabilities by eliminating interfacial water through rapid hydration. Yuk et al. designed a dry tape that adhered to tissues, achieving chemical bond formation between the NHS ester and tissue functional groups [33]. Powdered adhesives have also garnered attention for rapid water removal [149, 150, 151, 152]. Peng et al. prepared a self‐gelling powder combined with PEI and PAA, which could absorb interfacial water [11]. This powder could physically crosslink gel in situ within 2 s, exhibiting strong adhesion enhanced by the rapid removal of interfacial water between the hydrogel and the tissue. In addition, this hydrogel established topological entanglements with tissues through polymer physical diffusion for stable adhesion, effectively sealing the damaged porcine stomach and intestinal tissues. Similarly, Guo et al. prepared hybrid dry powders (HDPs) consisting of equal amounts of dry polycationic and polyanionic powders [10]. HDPs removed interfacial water rapidly, enabling electrostatic and hydrogen bonding interactions for immediate wet adhesion within seconds, and further enhanced adhesion by polymer physical diffusion. These HDPs achieved about 30 kPa adhesion strengths on wet porcine skin.

Mussels secrete Mfps composed of both proteinaceous components and hydrophobic amino acids. These Mfps form hydrophobic microdomains at the adhesion interface that actively displace interfacial water, creating locally dry conditions. Subsequently, the DOPA within these proteins demonstrates exceptional wet adhesion capabilities through synergistic interfacial interactions, including hydrogen bonding, metal coordination, and π‐cation interactions [153, 154, 155].

Inspired by mussel adhesion mechanisms, hydrophobic organic solvents, monomers, and polymers are used to develop molecular‐level (∼nm) dehydration strategies to efficiently remove hydration layers and enhance surface wetting. For instance, Yuk et al. prepared a barnacle‐glue‐inspired paste employing a hydrophobic oil matrix to exclude interfacial water, thereby creating favorable conditions for bioadhesive microparticles to establish contact with tissue surfaces [53]. Cui et al. developed a hydrophobic polymer‐based system for achieving robust wet adhesion through interfacial water displacement [94]. This unique hyperbranched polymer adhesive (HBPA) was prepared by ternary Michael addition of pentaerythritol tetraacrylate, short‐chain PEGDA, and dopamine hydrochloride. HBPA rapidly contracts upon contact with water, expelling interfacial water and exposing the Cat groups to achieve rapid adhesion. The adhesion strength to bone could reach up to 270 kPa. Utilizing hydrophobic association and supramolecular interactions, Yang et al. removed the hydration layer and achieved underwater adhesive [156]. This hydrogel was synthesized through the copolymerization of hydrophobic monomers (hexyl acrylate and styrene) and hydrophilic AA, further enhanced by a bifunctional host/guest system of β‐cyclodextrin (CD) and adamantane. Hydrophobic domains at the hydrogel surface remove water and build a hydrophobic environment to minimize interference from water. Concurrently, hydrophilic components facilitate adhesion via hydrogen bonding, electrostatic interactions, and cation–π coordination. In summary, addressing the problems of adhesion in the wet GI environment has led to advancements in hydrogel adhesives, largely enhancing their capacity to adhere securely and presenting promising solutions for GI perforation repair.

#### 2.3.2 Adhesion Under Acidity and Digestive Enzymes

**FIGURE 10:**
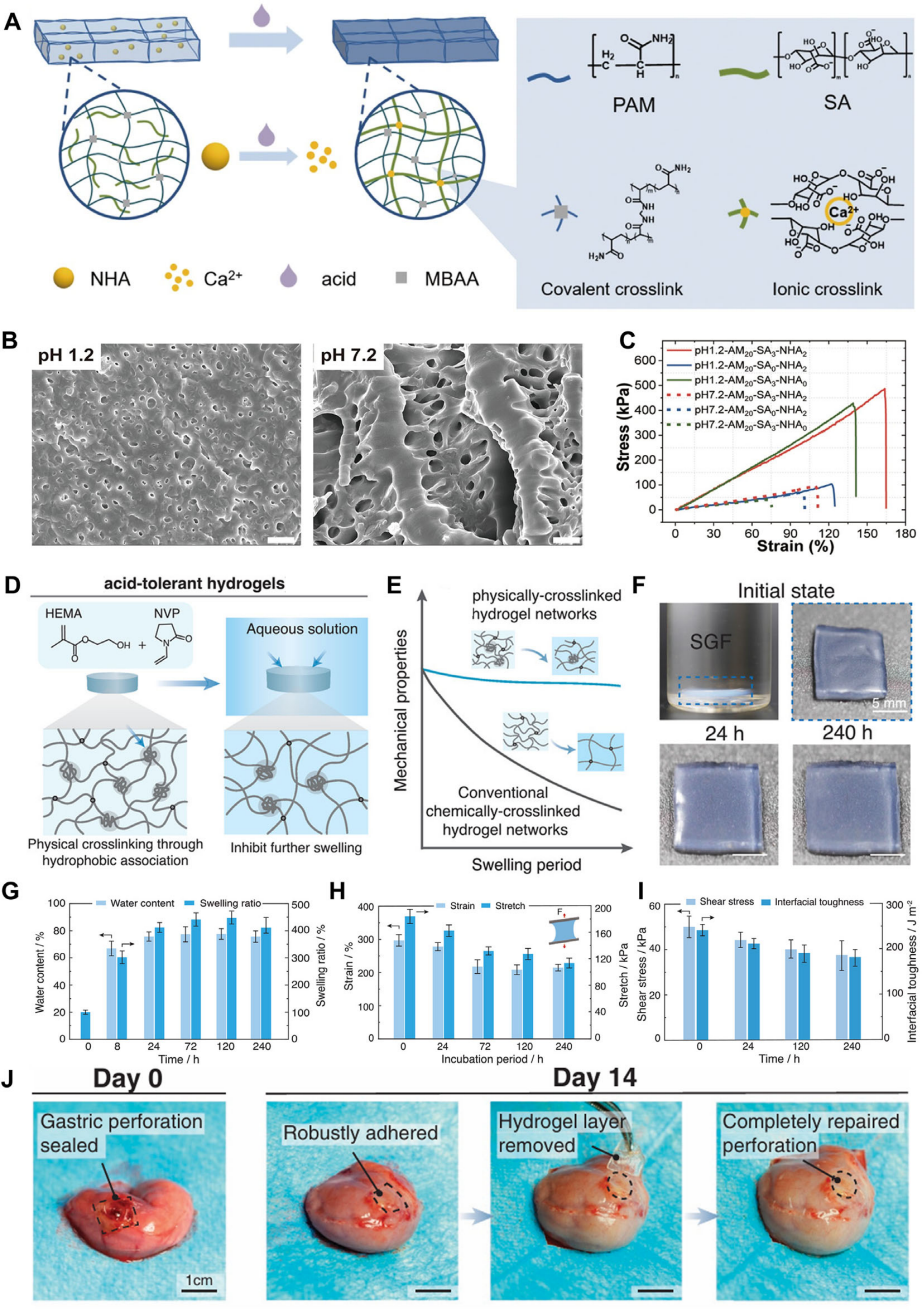
The applications of hydrogel adhesives in acidic environments. (A) Schematic of the preparation of PAM–SA–NHA hydrogel. (B) The scanning electron microscope (SEM) images of PAMSA–NHA hydrogels treated with pH 1.2 and pH 7.2. The structure of the hydrogel was more compact in an acidic environment. Scale bar: 5 µm. (C) The tensile stress–strain curves of PAM–SA–NHA hydrogels at different formulations and different pH levels. The mechanical properties of the hydrogel were stronger under an acidic environment. Reproduced with permission from reference [163]. Copyright 2021, Springer Nature. (D) Schematic illustration of limiting hydrogel further swelling and (E) hydrogel mechanical strength loss by hydrophobic interaction in the ATGels network. (F) Photographs of ATGels placed in a simulated gastric fluid medium at different times. Scale bar: 5 mm. (G) ATGels reached the swelling equilibrium after 24 h. (H) The strength of ATGels still exceeded 100 kPa after 240 h of immersion in simulated gastric fluid. (I) The toughness of ATGels adhered to the pigskin was able to exceed 200 J m^−2^ after 240 h of immersion in the simulated gastric fluid medium. (J) Photographs of gastric perforation on day 0 and the repaired stomach after 14 days. On day 14, the ATGels remained adhered to the stomach, and the perforation was completely sealed. Scale bar: 1 cm. Reproduced with permission from reference [19]. Copyright 2022, John Wiley and Sons.

It is well established that the stomach has an acidic environment with a pH of about 1–3, and the healthy intestines have a pH of about 6.4–7.5 [157, 158]. Notably, UC presents with a pH of approximately 5.5 [159]. The variability and extremity of these pH conditions pose challenges for the adhesion of hydrogel adhesives in the GI tract. Meanwhile, an excessively acidic environment can disrupt the hydrogel, decreasing mechanical strength and adhesion strength, causing undesirable swelling, and shortening retention time in the stomach, thus affecting the repair of the GI perforations [19]. For example, although Schiff base‐based adhesives can be applied in humid environments, the stability of these bonds decreases with lowering pH [160]. The development of acid‐resistant hydrogels has facilitated the application of hydrogel adhesives for repairing GI perforations [161]. Chemically crosslinked hydrogels are generally recognized for their superior integrity and acidic stability in the stomach. He et al. prepared a series of novel injectable pH‐responsive hydrogels by combining acryloyl‐6‐aminocaproic acid (AA), AA–g–N–hydroxysuccinimide (AA–NHS), and *N*, *N*′‐methylenebisacrylamide as a chemical crosslinker to accelerate gastric hemostasis and wound healing [58]. This hydrogel adhered through the formation of chemical bonds between ─NHS esters and ─NH_2_ on tissues, enhanced by hydrogen bonding interactions. In addition, based on chemical bond crosslinking, the AA/AA–NHS hydrogels exhibited low swelling under acidic conditions and minimal degradation during the period of usage. Beyond utilizing chemical crosslinkers, the pursuit of enhanced hydrogel stability in the GI tract necessitates the exploration of alternative methods. Notably, adjusting the composition and structure of hydrogels represents a promising alternative strategy to achieve this goal. Multi‐network hydrogels, known for their robust mechanical properties, can mitigate undesirable swelling in GI perforation repairs [119, 162]. Yuan et al. developed a dual‐network hydrogel by adding nano‐hydroxyapatite (NHA) as an ionic nano‐reservoir [163]. As shown in Figure 10A, they mixed acrylamide (AM), NHA, and SA solutions; used light‐controlled adhesive patch (LAP) as photoinitiator; and *N*, *N*'‐methylenebisacrylamide as a chemical crosslinker to prepare such a kind of dual network hydrogel, PAM–SA–NHA. The adhesion could be achieved through various interactions, including amide bonds formed by the activation of ─COOH by 1‐ethyl‐3‐(3‐dimethylaminopropyl) carbodiimide (EDC)/NHS reacting with tissue and hydrogen bonding interactions. In addition, the precursor fluid infiltrated the tissue and polymerized to form a topological interlocking structure. The slow release of Ca^2+^ from NHA in an acidic environment formed a new second network with SA, inhibiting the dissolution of the hydrogel in gastric juice. Therefore, this hydrogel showed a more compact structure and stronger mechanical strength at pH 1.2 compared to pH 7.2 (Figure 10B,C). Continuous efforts of stable hydrogel adhesives have been made in recent years, of which Chen et al. further refined the concept by developing an acid‐tolerant hydrogel (ATGels) bioadhesive [19]. This hydrogel was composed of two parts: acid‐resistant hydrogel and adhesive hydrogel. Acid‐resistant hydrogels were prepared by hydroxyethyl methacrylate (HEMA), *N*‐vinylpyrrolidone (NVP), Irgacure 2959, and crosslinker PEGDA, termed poly(HEMA–NVP) hydrogel. Subsequently, this hydrogel film was then immersed in acrylic acid–NHS solution to introduce NHS groups and enhance the adhesion of ATGels. The hydrophobic interactions within the hydrogel inhibited the swelling and made it stable under acidic conditions (Figure 10D–F). The swelling ratio, tensile properties, and tissue adhesion strength of ATGels remained stable in the simulated gastric fluid medium (Figure 10G–I). ATGels could seal rat gastric perforation quickly, and there was no leakage of gastric fluid after 14 days of treatment (Figure 10J).

Moreover, the digestive tract contains many enzymes, such as pepsin and enteropeptidase, that play crucial roles in food digestion and decomposition. These enzymes can significantly affect the stability and structure of hydrogels, especially for amino acid‐based or peptide hydrogels. In this harsh environment, the stability of hydrogels for tissue repair is critical, requiring them to maintain a controlled solubility and degradation rate that aligns with wound healing progress [164]. Acidity and enzyme‐resistant hydrogels serve as a physical barrier to prevent GI perforation from gastric acid and pepsin stimulation, thereby promoting wound healing [165].

#### 2.3.3 Dynamic Movement

The GI tract undergoes various motions, such as peristalsis, segmentation, haustration, and mass movement, during the food digestion and absorption process. Consequently, the hydrogel adhesive may shift due to these movements, resulting in seal failure. In addition, the GI tract periodically undergoes food entering and emptying, along with the presence of digestive juices and food residues, which may cause deformation and abrasion of the adhesive through friction. An ideal hydrogel for GI perforation repair should possess robust adhesion capabilities to ensure a stable position and minimize the risk of migration [166]. Furthermore, it should also have strong mechanical properties to withstand the dynamic movement of the GI tract and prevent sealing failures due to material breakage. Current research efforts have been devoted to the development of hydrogels with high adhesion and mechanical strength [167]. Although most hydrogel adhesives are capable of withstanding bursting pressure (defined as the minimum pressure required to burst a sealing defect) and GI mechanical pressures ranging from 5 to 10 kPa in isolated porcine stomach models [148, 168, 169], achieving a stable seal within the GI tract for more than 48 h remains a challenge [16]. To address this, new chemical engineering strategies are being explored to develop hydrogels with enhanced mechanical resilience.

For adhesives adhering primarily through chemical bonds, those incorporating energy‐dissipating dynamic bonds, such as Schiff base linkages and disulfide bonds, exhibit enhanced durability and stability. Conversely, adhesives relying on NHS ester linkages cannot recover once hydrolyzed and lack dynamic stability. For example, Ge et al. developed a structurally dynamic self‐healing hydrogel through the host‐guest interaction between β‐cyclodextrin and amantadine for improved UC treatment [170]. This hydrogel was prepared by combining dopamine (DA)/β‐cyclodextrin (CD)‐modified HA (HA–CD–DA) and amantadine‐modified carboxymethyl chitosan (CMCS–AD) solutions and loading them with basic fibroblast growth factor (bFGF) and L‐alanyl‐L‐glutamine (ALG). This hydrogel, HCD–bFGF–ALG, demonstrated good adhesive capability, adhering to colon walls under constant PBS flow and stretched or twisted conditions. Furthermore, HCD–bFGF–ALG exhibited superior mechanical properties compared to natural intestinal tissues, featuring a Young's modulus of approximately 3 MPa and a break strain of about 75%. It achieved complete self‐healing within 1 minute post‐damage and could withstand continuous intestinal motility upon application. When injected into the mouse intestine via rectal enema, over 89.6% of the hydrogel remained in the colon of mice with UC after 24 h. Animal experiments utilizing fluorescently labeled hydrogels further demonstrated stable attachment at the rectal enema site, with persistence for up to 5 days.

Other hydrogels, including dual network hydrogels and homogeneous networking hydrogels, also exhibit preferable mechanical elasticity and fatigue resistance [171]. Notably, tetra‐arm poly(ethylene glycol)‐based hydrogels, resembling an ideal network with uniformly arranged macromolecules, possess a remarkably high maximum breaking strength of ≥27 MPa [172]. Xu et al. prepared more uniform crosslinked hydrogels by efficient equimolar reaction between thiourea groups and Cat, mitigating uncontrolled self‐polymerization within Cat‐based hydrogels [43]. This hydrogel adhesive maintained stable adhesion in an acidic gastric juice environment by proper oxidation of Cat groups and demonstrated adherence to ulcer sites for at least 48 h in the porcine gastric ulcer adhesion test, highlighting its potential for GI perforation repair.

## 3 The Applications of Hydrogel Adhesives for GI Perforations

The primary objective of hydrogel adhesive repair for GI perforations is to effectively seal the wound. However, given the complexity of disease pathology, the introduction of biological features into therapeutic strategy is crucial for accelerating wound healing and ensuring postoperative safety. Therefore, this section discusses the advancements in hydrogel adhesives that not only seal the wound but also modulate the microenvironment of GI wounds and prevent postoperative tissue adhesion.

### 3.1 Sealing of GI Perforation

**FIGURE 11:**
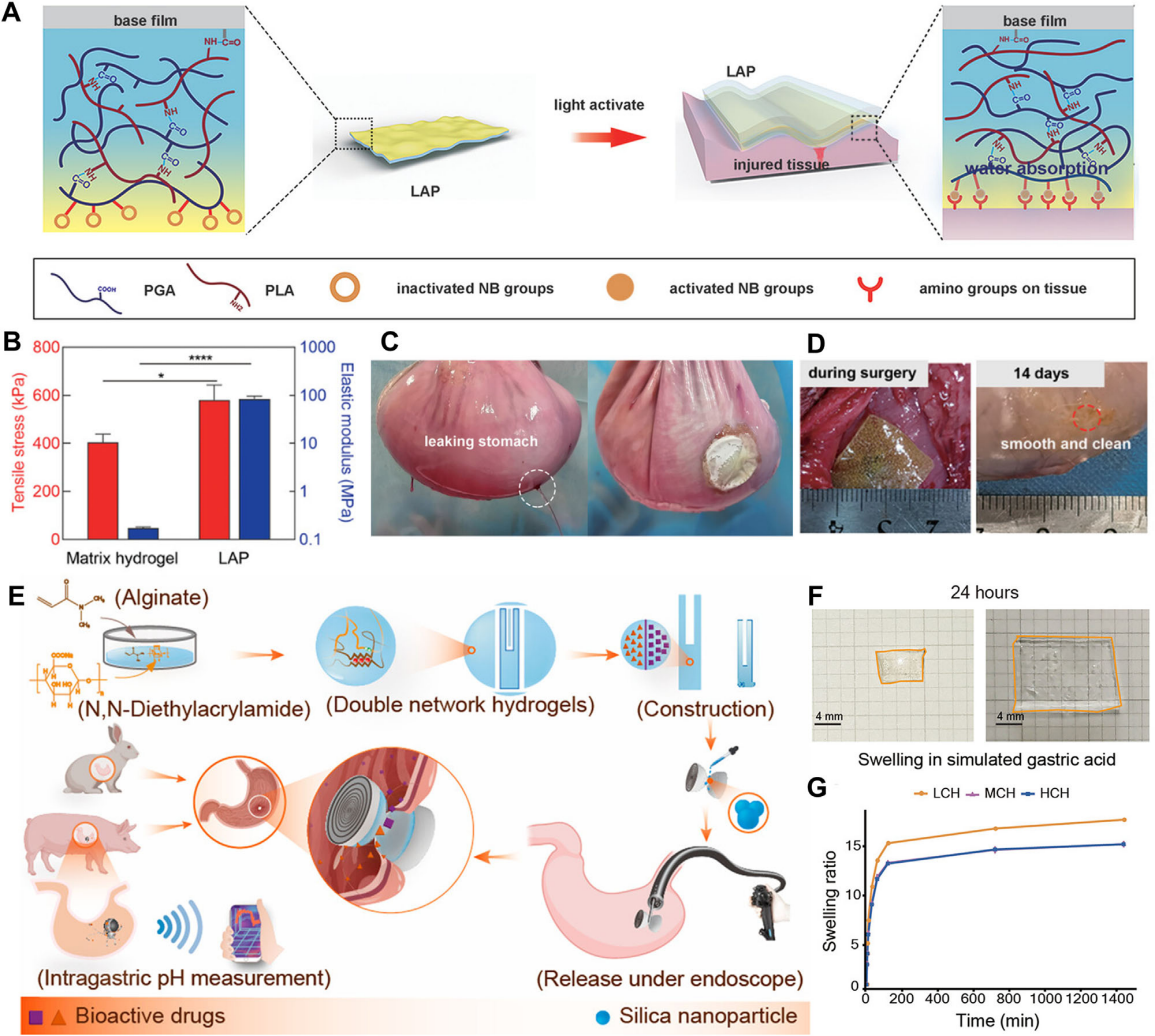
The applications of hydrogel adhesives for sealing GI perforations. (A) Schematic of the preparation of LAP hydrogel. (B) Mechanical properties of LAP hydrogel. (C) Photographs of the successful sealing of a liquid‐filled stomach by LAP. (D) Photographs of a rabbit gastric perforation model treated with UV–LAP. Reproduced with permission from reference [173]. Copyright 2022, John Wiley and Sons. (E) Schematic of the preparation of a dual networked, onion‐shaped hydrogel, which can be delivered through an endoscope. (F) The photographs and (G) swelling ratio of the hydrogel after immersion in simulated gastric fluid. Reproduced with permission from reference [148]. Copyright 2022, American Chemical Society.

Achieving rapid and stable sealing requires hydrogel adhesives with excellent adhesion properties. Zhu et al. prepared a tough LAP for non‐suture regeneration of visceral wounds [173]. This matrix hydrogel patch was prepared by incorporating NHS and EDC into the mixed solution of PGA and PLA, followed by vigorous stirring, and poured onto poly(L‐lactic acid) in a glass mold. The subsequent addition of *N*‐(2‐aminoethyl)‐4‐(4‐(hydroxymethyl)‐2‐methoxy‐5‐nitrosophenoxy) butanamide resulted in the formation of LAP (Figure 11A). Notably, the tensile strength of LAP reached 580.63 kPa, surpassing the maximum tension that pig stomach smooth muscle can endure by 104 kPa (Figure 11B). Upon activation with 405 nm UV light, the adhesion strength peaked at 143.1 kPa. Figure 11C shows that the robust adhesive strength, based on Schiff base bonds, and mechanical properties allowed LAP to fully seal pig stomachs with 5 mm holes even when filled with water. In a rabbit gastric perforation model, the surface of the defect was smooth and healed well after 14 days of treatment (Figure 11D), indicating satisfactory healing. Moreover, appropriate swelling of hydrogels in acidic environments can also effectively seal GI perforations. In another study, Liu et al. designed a dual networked, onion‐shaped hydrogel for the physical sealing of gastric perforations [148]. This hydrogel was prepared by mixing *N*, *N‐*dimethylacrylamine, SA, calcium carbide, initiator ammonium persulfate (APS)/*N*, *N*, *N*′*, N*′‐tetramethylenediamine, and crosslinker cystamine bis(acrylamide) (Figure 11E). With simple clipping and processing, this hydrogel device expanded like a mushroom and achieved a swelling ratio exceeding 15 in pH 1 simulated gastric fluid, which could successfully seal the gastric perforation by its swollen, onion‐like multilayer hydrogel device (Figure 11F,G). Notably, the hydrogel maintained stability in acidic conditions while gradually degrading over the treatment period. In addition, this adhesive, loaded with vonoprazan fumarate, a potassium‐competitive acid blocker, effectively elevated stomach pH above 4 for approximately 60 h, thereby facilitating wound healing.

### 3.2 Regulating the GI Perforation Microenvironment

**FIGURE 12:**
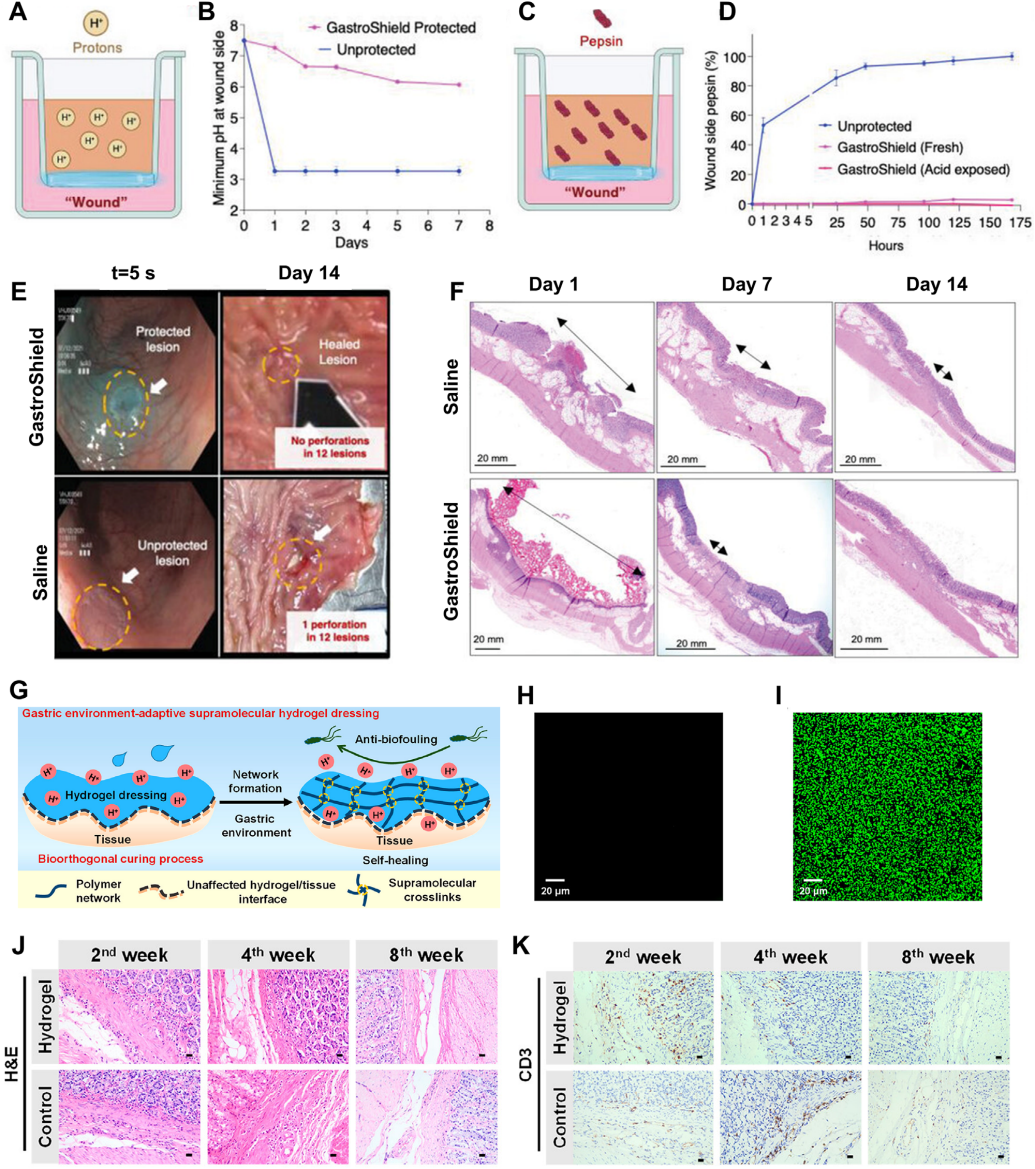
The applications of hydrogel adhesives in modulating the microenvironment of GI perforation. (A) Schematic illustration of simulated GastroShield wound protection from an acidic environment in vitro using the transwell system. (B) pH within the two chambers of the transwell system. Simulated gastric fluid was added to the upper chamber and PBS to the lower chamber. GastroShield was able to protect the cells from the acidic environment for at least one week. (C) Schematic illustration of simulated GastroShield wound protection from pepsin in vitro using the transwell system. (D) Pepsin concentration within the two chambers of the transwell system. Pepsin was added to the upper chamber. GastroShield blocked more than 98% of pepsin. (E) Photographs of endoscopic application of different treatments in vivo. (F) H&E staining of gastric perforation tissues at different time points. Scale bar: 20 mm. Arrows indicate lesion size. Reproduced with permission from reference [174]. Copyright 2024, John Wiley and Sons. (G) Schematic illustration of the role of ANGNA hydrogel. Fluorescence staining images of *E. coli* on (H) hydrogel coating and (I) glass. The hydrogel coating resists contamination by *E. coli*. Scale bar: 20 µm. (J) H&E staining and (K) immunohistochemical staining for CD3 of tissues after treatment with conventional omental implantation (Control) and hydrogel in a rat gastric perforation model. Scale bar: 20 µm. Reproduced with permission from reference [125]. Copyright 2021, American Chemical Society.

A bicarbonate mucus barrier protects the healthy stomach lining from damaging effects, including gastric juices and digestive enzymes, thereby preventing erosion. Disruption of this barrier leads to gastric microenvironment disorganization, erosive gastritis, ulcers, and ultimately gastric perforation, prompting research into hydrogel adhesives with barrier properties. Taboada et al. proposed a novel sprayable bioadhesive hydrogel, GastroShield, formulated with oxidized dextran and PEI‐modified Pluronic‐F68 [174]. The sealing and proton sponge capabilities of GastroShield protected the monolayer of L929 cells from simulated gastric fluid (pH 2) and pepsin (1.8 mg mL^−1^) in a transwell system. Notably, such a hydrogel protected the cells from gastric acid for more than a week (Figure 12A,B) and blocked 98% of pepsin permeation (Figure 12C,D), attributed to PEI groups functioning as proton sponges. It served as an effective physical barrier against hydrogen ion permeation, which was pivotal for its scientific and technological applications. In addition, the hydrogel exhibited strong tissue adhesion based on interdigitation, ionic and electrostatic interactions, and covalent imine bonds, maintaining burst pressures of 64.7 ± 11 mmHg at t_0 h_ and 56.3 ± 12 mmHg at t_24 h_. When administered endoscopically to porcine gastric wounds, this material achieved full wound coverage within 5 s (Figure 12E) without off‐target spraying or dripping, protecting the GI wound microenvironment. Figure 12F shows that GastroShield accelerated gastric perforation healing compared to saline.

Although hydrogel adhesives can act as a barrier, bacterial infections in the microenvironment that arise from gastric content leakage and dysbiosis need to be addressed. Antibiotic administration is a routine treatment to minimize complications for gastric perforation bacterial infections [175, 176]. However, long‐term use of antibiotics causes bacterial resistance, disrupts intragastric flora homeostasis, and affects the treatment effectiveness. Zhao et al. developed a nonantibiotic, anti‐infective therapeutic patch designed to modulate the electrical microenvironment of biofilms adjacent to GI wounds [177]. In conjunction with extracorporeal ultrasound therapy, this patch disrupted the biofilm's electron transport chain, suppressed bacterial proliferation, and exhibited antibacterial infection activity in a rat cecum puncture model. Furthermore, Wang et al. prepared an antifouling hydrogel, ANGNA, through the synthesis of an ABA block polymer by PEG, poly(*N*‐isopropyl acrylamide), and pH‐sensitive acryloyl‐6‐aminocaproic acid (A6ACA) [125]. The protonated A6ACA within this hydrogel adheres to tissue surface ─NH_2_ and ─COOH via hydrogen bonding interactions. Under acidic conditions, ANGNA hydrogels formed hydrogels by supramolecular crosslinking and achieved antifouling properties through PEG hydrophilic blocking (Figure 12G). The PEG hydrophilic layer formed acts as a barrier against *E. coli* adhesion. As shown in Figure 12H,I, the hydrogel reduced bacterial adhesion. In rat gastric perforation models, the anti‐fouling hydrogel dressing inhibited the inflammatory response and decreased T cell inflammatory infiltration (Figure 12J,K).

In addition to bacterial infection, the impaired blood vessels hinder mass transfer in the microenvironment of the GI perforations. Activated pepsin in an acidic environment degrades regenerative factors that promote neovascularization, delaying wound healing [178, 179]. To address the abnormal expression of growth factors, Shanmugapriya et al. incorporated an epidermal growth factor receptor to maintain a conducive environment suitable for GI perforation healing [180]. However, reliance on growth factor regulation alone may be insufficient due to the complex interplay of factors involved in the healing process. For example, the inflammatory response triggered by GI damage results in a significant increase in reactive oxygen species (ROS) within the microenvironment. This elevation leads to oxidative stress and disrupts the redox balance, further impeding the healing of GI perforations [181]. Therefore, researchers loaded ROS‐sacrificing components into the hydrogel to scavenge ROS, improve the microenvironment, and accelerate wound healing [182, 183]. Ni et al. have reported a novel hydrogel adhesive tailored for gastric ulcer repair, which scavenges ROS at the wound site via lactobionic acid, a polyhydroxy organic acid with potent antioxidant properties [184]. This hydrogel showed the ability to scavenge 1,1‐diphenyl‐2‐picrylhydrazyl, 2,2′‐azino‐bis(3‐ethylbenzothiazoline‐6‐sulfonic acid), 2‐phenyl‐4,4,5,5‐tetramethylimidazoline‐3‐oxide‐1‐oxyl, hydroxyl radicals, and hydrogen peroxide in vitro tests. It also alleviated oxidative stress, promoted epithelialization and angiogenesis in a rat gastric ulcer model, and effectively facilitated the healing of gastric ulcers.

### 3.3 Preventing Intraperitoneal Adhesion After GI Perforation Repair Surgery

Intraperitoneal adhesions arising from post‐digestive surgical procedures pose a significant clinical challenge, constituting traumatic scars and adhesions between two normally unattached adjacent peritoneal surfaces due to abnormal peritoneal formation [185]. These adhesions cause pain, hinder subsequent surgeries, and complicate patient recovery from GI perforations [186]. While the previously mentioned DST exhibits excellent adhesion properties, there is a tissue adhesion risk when DST is used individually, so it is often combined with backing layers or other hydrogels [33]. These limitations have motivated the emergence of Janus hydrogel adhesives, which feature precisely engineered asymmetric architectures or interfacial heterogeneities, marking a technological advancement for achieving simultaneous tissue sealing and adhesion prevention in internal tissue repair [187, 188]. Existing research had engineered Janus functionality in hydrogel adhesives by constructing bilayer architectures. Yang et al. designed a PAA‐NHS‐based sandwiched patch for sealing intestinal injuries [189]. The inner adhesive layer adheres firmly to the intestinal wound by chemical bonds, and the bursting pressure can reach 21 kPa after intestinal sealing. On the other side of the patch, an anti‐adhesion layer is prepared based on zwitterions to resist cell and protein adhesion. However, such bilayer‐structured hydrogels not only require multistep fabrication processes but also exhibit distinct interfaces between layers that are prone to mechanical delamination during tissue movement.

In contrast, Janus hydrogel adhesives with seamless macroscopic interfaces have attracted considerable attention due to their structural integrity [190]. Cui et al. prepared a carboxylate‐rich wet adhesion hydrogel by introducing a cationic chitooligosaccharide (COS) into the hydrogel [20]. The interaction between COS and ─COOH groups triggered phase separation of the polyelectrolyte complexes near the hydrogel's bottom surface, which reduced the interfacial energy and increased the hydrophobicity. At the same time, the bottom surface of the hydrogel was white and hydrophobic, exposing a large number of ─COOH to achieve adhesion to soft wet tissues through hydrogen bonding interactions, promoting tissue repair. However, the ─COOH on the opposite side, tightly bound to the COS, lost its adhesion ability, making it a Janus hydrogel. With a similar principle, Liang et al. also prepared a Janus hydrogel adhesive for the gastric perforation treatment [191]. This hydrogel achieved asymmetric adhesion, avoiding adjacent tissue adhesion and accelerating the healing of the gastric perforation compared with omentum majus.

To further simplify the fabrication processes, a variety of Janus hydrogel adhesives have been extensively developed through “one‐pot” synthesis strategies, including interface modification [192], gravity and buoyancy‐driven separation [193], and other approaches [194]. Among these, Wang et al. developed an integrally formed Janus hydrogel via stirring‐induced emulsion phase separation coupled with in situ polymerization [195]. In this hydrogel, low‐density emulsion droplets accumulated at the upper surface to form a hydrophobic physical barrier while simultaneously restricting ─COOH group exposure to reduce tissue bonding on this side. The resulting adhesive strength differential between the two surfaces reached a 20‐fold contrast. These droplets subsequently served as hydrophobic physical crosslinks to enhance mechanical properties. When evaluated in stomach defect models, the adhesive demonstrated rapid tissue adhesion within 30 s on wet wound surfaces, withstood pressures up to 362 mmHg, and promoted gastric wall regeneration within 14 days while effectively preventing postoperative adhesions with the omentum and liver.

Similarly, Fang et al. fabricated Janus particles (JPs) @ PAA‐polyurushiol (PU) Janus hydrogels through UV‐initiated one‐pot polymerization of PAA and PU phases stabilized by JPs [196]. The hydrophobic PU phase spontaneously migrated upward to form a barrier layer, while the PAA‐rich bottom layer provided tissue adhesion. The structural integrity of this interface is maintained through a mercaptoene click reaction between the SiO_2_ knob of JPs and double bonds within the hydrogel matrix, effectively stabilizing the oil–water interface. The adhesive interface formed multiple bonds with tissues, including hydrogen bonding, electrostatic interactions, mechanical interlocking, and covalent crosslinking through exposed ─COOH groups. Meanwhile, the PU‐dominated upper layer, characterized by its hydrophobic long alkyl chains and benzoquinone structures, created an effective barrier against blood extravasation and protein adsorption. Such a Janus hydrogel adhesive shows the prospect of clinical operation, making it a promising choice for in vivo applications.

## 4 Clinical Applications of Hydrogel Adhesive in Repairing GI Perforation

### 4.1 Clinically Approved Hydrogel Adhesives

Hydrogel adhesives, such as fibrin glue (TISSEEL), albumin‐based adhesive (BioGlue), and PEG‐based adhesive (DuraSeal), have been clinically applied in repairing GI perforations [197, 198, 199]. Some commercial hydrogel adhesives are shown in Table 2. Fibrin glue is a protein‐derived product extracted from human blood, which consists of part A, rich in fibrinogen and factor XIII, and part B, with calcium ions and thrombin. It simulates the end stage of the coagulation cascade and has been used to prevent leakage from colonic anastomoses following the reversal of temporary colostomies [200, 201]. Factor XIII, a fibrin‐stabilizing factor, can be activated by thrombin to form factor XIIIa. In the presence of Ca^2+^, factor XIIIa promotes a network of stabilizing cross‐links of fibrin monomers in the form of amide bonds between glutamine and lysine residues [202, 203]. Fibrin glue achieves tissue adhesion by adsorbing erythrocytes and platelets to form blood clots, through hydrogen bonding and topological linkages. It can be completely reabsorbed by macrophages and fibroblasts within two weeks after application [204, 205]. In addition, its dual components make it injectable, facilitating delivery via endoscopy. Many research works have utilized fibrin sealants for the management of postoperative wounds and gastrodermal fistulae [205, 206, 207]. However, the application of fibrin glue requires adjunctive suturing for optimal wound closure.

**TABLE 2:** Representative commercial hydrogel adhesives for clinical applications.

Commercial products	Composition	Applications	Ref.
TISSEEL	Fibrinogen and factor XIII Calcium ions and thrombin, and contains aprotinin	Hemostasis, sealant, closure of fistulae, and anastomotic leaks	[198, 199, 200, 201, 214]
Beriplast P	Human fibrinogen, coagulation factor XIII (human), aprotinin (bovine lung) Human thrombin, calcium chloride	Closure of duodenal perforation	[208]
Crosseal	Human plasma A highly purified preparation of human thrombin (without aprotinin)	Hemostasis, sealant	[197, 204]
Hemaseel	Human plasma A highly purified preparation of human thrombin (without aprotinin)	Hemostasis, sealant	[204]
Evicel	Human clottable protein (predominantly human fibrinogen) Human thrombin (without the antifibrinolytic agent tranexamic acid and aprotinin)	Hemostasis, suture support	[197]
Quixil	Similar to Tisseel, it contains the antifibrinolytic agent tranexamic acid	Hemostasis	[197]
BioGlue	45% purified bovine serum albumin 10% glutaraldehyde solution	Sealing cardiovascular	[21, 211, 212, 213, 214, 215]
PreveLeak	Purified BSA Polyaldehyde	Sealing cardiovascular	[211]
DuraSeal	A PEG ester solution A triphenylamine solution	Dural repair, closure of duodenal perforation	[21, 199, 200, 201, 202, 203, 204, 205, 206, 207, 208]
CoSeal	Two synthetic PEGs A dilute hydrochloric acid solution A sodium dihydrogen phosphate/sodium carbonate solution	Sealing cardiovascular and GI perforation	[21, 199, 211, 216]
Tridyne VS	PEG Human serum albumin	Sealing cardiovascular	[211]

In addition, DuraSeal sealant, composed of a PEG ester solution and a triphenylamine solution, is considered a replacement for fibrin glue [208]. In the repair of duodenal perforation, DuraSeal exhibits similar sealing effectiveness to fibrin glue and can even withstand higher pressures. Another PEG‐based sealant, CoSeal, which consists of two synthetic PEGs, a dilute hydrochloric acid solution, and a sodium dihydrogen phosphate/sodium carbonate solution, is FDA‐approved for sealing blood vessels. However, Taboada et al. showed that Coseal exhibited a reduction in burst pressure by 89% after 24 h [174]. Yang et al. used CoSeal to treat intestinal injuries, and the results showed that its adhesive strength was insufficient to effectively seal bleeding intestinal tissue, and visual postoperative tissue adhesions were caused after 2 weeks postoperatively [189]. The aforementioned hydrogel adhesives require mixing of the two components, and the limited speed of gelation and mechanical and adhesive properties limit the independent use of adhesives such as fibrin glue, which necessitates constant postoperative observation, repeated injections of fibrin sealant, or use with metal clips to ensure wound closure [209].

BioGlue consists of 45% purified BSA in a 10% glutaraldehyde solution [210] and showed significantly stronger arterial burst test results compared to Coseal [33, 211]. However, unpolymerized BioGlue harmed the survival of HMEC‐1 cells. In addition, a variant of the fibrin sealant Vitagel, the Gel–thrombin matrix sealant Floseal, is also commonly used clinically as an adhesive [212, 213].

### 4.2 Clinical Trial‐Stage Hydrogel Adhesives and Translational Challenges

#### 4.2.1 Clinical Trial‐Stage Hydrogel Adhesives

As shown in Table 3, some other hydrogel adhesives have advanced to the clinical trial stage. Hydrogels, as a promising drug delivery vehicle, enable controlled and targeted drug release. Among these, bioactive component‐loaded adhesives, such as those incorporating purified exosome product (PEP) and platelet‐rich plasma (PRP), have also been implemented in clinical investigations. For example, Khan et al. conducted a clinical study evaluating the therapeutic efficacy and safety profile of a PEP‐TISSEEL composite for treating chronic radiation‐induced ulcers (NCT06793748). Shi et al. investigated the clinical outcomes of PRP combined with thrombin gel, focusing on both safety parameters and treatment effectiveness (NCT05912712). For acute GI bleeding management, Eruchalu et al. performed a comparative clinical trial assessing UI‐EWD (Nexpowder) hemostatic powder versus conventional endoscopic hemostatic therapies (NCT06188585). In the oncology domain, Zhang et al. developed a thermosensitive hydrogel loaded with 5‐fluorouracil and systematically evaluated its safety and antitumor efficacy in colorectal cancer treatment (NCT06385418). Furthermore, Kwon et al. designed a randomized controlled trial to determine whether ropivacaine‐incorporated poloxamer 407 hydrogel provides non‐inferior postoperative pain control compared to a transversus abdominis plane block in patients undergoing minimally invasive gastrectomy (NCT06839716). The aforementioned in‐trial products are basically in the early stage of clinical research, which needs further validation for clinical translation.

**TABLE 3:** Representative hydrogel adhesives for GI repairing in the clinical trial stage (data from ClinicalTrials.gov).

NCT ID	Hydrogel adhesive	Phase and location	Disease models
NCT06793748	PEP^TM^, fibrin sealant	Phase 1/2, United States	Chronic radiation ulcer
NCT05912712	Autologous PRP, thrombin coagulum gel	Phase 1, China	Topical treatment of rectal mucosal ulcers
NCT06188585	UI‐EWD composed of aldehyde dextran and succinic acid–modified ε‐poly [217]	Not applicable, Canada and Denmark	Acute overt GI bleeding
NCT06385418	Fluorouracil, poloxamer 407, and poloxamer 188	Phase 2, China	Colorectal cancer
NCT06839716	Ropivacaine, poloxamer 407‐based gel (Welpass)	Not applicable, Republic of Korea	Minimally invasive gastrectomy

#### 4.2.2 Challenges in the Material Properties of Adhesives

Hydrogel adhesives face challenges in their material properties for GI applications due to the harsh biological environment, which features wetness, digestive fluid erosion, and dynamic mechanical stress. Human intestinal peristalsis occurs at approximately 10 cycles per minute, accompanied by active contact forces of about 0.18–0.5 N cm^−1^, periodic radial compressive pressures of about 20 mmHg, and shear stresses oscillating at 0.125–0.3 Hz with 1 N cm^−2^ [218, 219, 220, 221]. These dynamic forces demand hydrogel adhesives to simultaneously achieve interfacial adhesion and bulk mechanical properties. However, such a dual requirement presents the fundamental design challenge. Excessively rigid networks, for example, with high crosslinking densities, risk mechanical mismatch with soft intestinal tissues and subsequent interfacial delamination; insufficient cohesion may result in bulk fragmentation. Adhesives should be strategically designed to optimally balance between the two requirements. To effectively repair GI perforations, hydrogel adhesives need to meet performance criteria, including storage modulus >6 kPa, interfacial toughness >50 J m^−2^, burst pressure >120 mmHg, and robust wet adhesion with an adhesive strength >10 kPa for over 7 days [16]. These requirements ensure sufficient mechanical integrity and long‐term functionality under physiologically demanding conditions. Moreover, the biodegradation performance of adhesives needs to be tailored to the organ‐specific enzymatic microenvironment. For example, silk fibroin exhibits resistance to degradation by pepsin and has been successfully employed to seal gastric perforations [222]. Nevertheless, it is selectively degradable by enzymes in the small intestine, which is not conducive to the healing of intestinal wounds [223].

Moreover, as minimally invasive and ultra‐minimally invasive techniques advance as frontline approaches for GI perforation repair by preserving organ architecture, minimizing peritoneal trauma, and preventing postoperative adhesions, injectable hydrogels suitable for endoscopic delivery have emerged, necessitating precise control of their rheological properties [224, 225]. For example, in endoscopic delivery systems designed for injectable adhesives, the adhesive viscosity must be carefully controlled to prevent unintended leakage into non‐target areas caused by excessively low viscosity, which could lead to accidental adhesion. The influence of shear rate on viscosity must be thoroughly evaluated for the nearly 2‐meter long endoscope injection needle with an inner diameter of 2–3 mm [226]. For high‐viscosity adhesives, the required injection force should remain within clinically manageable limits. The mean downward thumb‐pushing strength is 184 N for males and 135 N for females aged 21–30 [227]. For injectable hydrogels, materials should allow smooth delivery of 5 mL substance under an injection force <12 N [228]. Furthermore, the hydrogel curing time must be synchronized with the endoscopic operation to ensure optimal clinical outcomes. Premature curing may result in incomplete wound coverage, whereas delayed curing could lead to adhesive migration to non‐target sites, ultimately compromising surgical success.

#### 4.2.3 Challenges of Supporting Devices

During the delivery of hydrogel adhesives for GI repair using endoscopic systems, the supporting devices and adhesive may interact with each other, leading to unintended consequences. For instance, the materials of spray catheters or injection needles may react with components in the hydrogel, altering its gelation time. Moreover, different types of hydrogel adhesives impose distinct functional requirements on the delivery devices. For thermosensitive hydrogels, the endoscope must traverse the GI tract, where temperatures normally vary [229]. To ensure proper gelation at the target site, the endoscope should be equipped with a precise temperature regulation system to maintain an optimal delivery temperature. When delivering powdered hydrogel adhesives, the focus shifts to accurate dosage control and spatial precision to prevent unintended dispersion, which could compromise repair efficacy. These challenges highlight the need for customized endoscopic accessories tailored to ensure reliable performance in clinical applications.

#### 4.2.4 Regulatory Challenges and Future Directions

Governments worldwide have strengthened regulatory oversight of pharmaceutical and medical products by establishing comprehensive technical review guidelines. These guidelines impose rigorous requirements for product registration, including clear labeling of indications and contraindications, detailed documentation of starting materials, final product structure, material sources, purity, dosage specifications, and supplier information. Manufacturers must also elucidate the functional roles and mechanisms of all adhesive components while conducting thorough technical validation of product performance.

For biologically derived adhesives like fibrin‐based hydrogels, enhanced quality control measures are mandated. This includes stringent monitoring of porcine blood storage conditions, cleanroom environmental parameters, microbial content, and batch‐to‐batch consistency in critical parameters such as clotting activity, purity, chemical residues, protein concentration, and enzymatic activity to ensure product reliability. Production processes must employ appropriate sterilization methods with compatible packaging solutions. Strict adherence to good manufacturing practice is essential throughout manufacturing to guarantee product quality, safety, and consistency through systematic quality management systems. Post‐marketing surveillance remains crucial, as evidenced by cases where surgical adhesives demonstrated incomplete polymerization or suboptimal bonding strength in clinical applications, potentially compromising patient safety in extreme scenarios.

While current regulatory frameworks provide guidance for GI perforation adhesives, significant limitations persist in existing standards. For instance, the standard test method for strength properties of tissue adhesives in lap‐shear by tension loading, ASTM F2255‐05, lacks provisions for dynamic wet adhesion testing, creating a performance validation gap. Furthermore, the biocompatibility of hydrogel adhesives has been evaluated mostly in accordance with relevant regulatory standards such as ISO 10993 and China's GB/T 16886 [230, 231]. However, current research on biocompatibility tests relies on non‐human cell lines and animal models, failing to adequately investigate the potential immunogenic responses that these hydrogel products may induce in humans. These methodological limitations hinder the comprehensive evaluation of product safety and efficacy in complex biological environments. A systematic evaluation framework should be established to address current limitations and enhance assessment standards for GI perforation sealants.

## 5 Conclusion and Outlook

Hydrogel adhesives have emerged as promising sealing materials for GI perforation repair due to their high similarity to native tissues, appropriate interfacial adhesion strength, excellent biocompatibility, and tunable mechanical properties. These advantages make them promising candidates to replace sutures and anastomotic staples in GI perforation repair surgery. This work summarizes both the adhesion mechanisms between hydrogels and tissue surfaces and representative applications of hydrogel adhesives in managing GI perforations. Among various hydrogel tissue adhesives, covalent bonding and noncovalent interactions often work synergistically to achieve robust adhesion. Covalent bonds, typically formed through tissue interactions involving NHS or SH functional groups, provide the primary mechanical strength required to seal GI perforations. Noncovalent interactions, on the other hand, are crucial during the initial stages of adhesion by facilitating rapid and intimate contact between hydrogels and the tissue surfaces. Hydrogen bonds can quickly form temporary attachments and fill gaps, allowing the hydrogel to conform closely to the tissue surface. Adhesion can be further enhanced through electrostatic interactions between charged groups on the hydrogel and tissue surfaces. The integration of covalent and noncovalent interactions is critical for achieving optimal adhesion. In addition to sealing performance, the ability to modulate the GI microenvironment and prevent postoperative tissue adhesion is also essential for clinical translation. Accordingly, certain multifunctional hydrogel adhesives not only provide effective sealing of GI perforations but also contribute to microenvironmental regulation and tissue healing, offering enhanced therapeutic potential and opportunities for clinical translation.

Despite significant advances, several challenges remain in the development and application of hydrogel adhesives in biomedical research and clinical practice. Firstly, the in vivo safety of synthetic polymer‐based hydrogels requires further validation. The use of chemical initiators, crosslinkers, and monomers in hydrogel synthesis may lead to the gradual release of small molecules, potentially compromising biocompatibility. This concern can be addressed through purification techniques such as dialysis, lyophilization, or reconstitution, which help eliminate residual impurities and improve biocompatibility. However, the long‐term in vivo biosafety and potential toxicity of most hydrogel adhesives remain largely unknown. Second, the development of hydrogel adhesives suitable for endoscopic delivery is urgently needed. Most current research focuses on film‐type hydrogel adhesives, which often require open abdominal surgery for application, thereby increasing the risk of infection and patient discomfort. As ultra‐minimally invasive endoscopic techniques become more prevalent in GI surgery, there is a growing need to develop hydrogel adhesives compatible with endoscopic delivery. Such systems could facilitate postoperative wound repair, reduce complications, and improve patient outcomes. Lastly, the complex synthesis process of hydrogel adhesives, which often involves multiple chemical reactions, presents a barrier to large‐scale production. Therefore, simplifying and standardizing fabrication protocols, improving manufacturing efficiency, and reducing costs are essential for clinical translation. Ultimately, the development of more effective, scalable, and user‐friendly hydrogel adhesives will enable the next‐generation strategy for managing GI perforations.

## Conflicts of Interest

The authors declare no conflicts of interest.

## Data Availability

Data sharing is not applicable to this article as no new data were created or analyzed in this study.
